# Magnetic fields reveal signatures of triplet-pair multi-exciton photoluminescence in singlet fission

**DOI:** 10.1038/s41557-024-01591-0

**Published:** 2024-07-25

**Authors:** Jiale Feng, Parisa Hosseinabadi, Damon M. de Clercq, Ben P. Carwithen, Michael P. Nielsen, Matthew W. Brett, Shyamal K. K. Prasad, Adam A. D. Farahani, Hsiu L. Li, Samuel N. Sanders, Jonathon E. Beves, N. J. Ekins-Daukes, Jared H. Cole, Pall Thordarson, David M. Huang, Murad J. Y. Tayebjee, Timothy W. Schmidt

**Affiliations:** 1 ARC Centre of Excellence in Exciton Science, School of Chemistry, UNSW Sydney, Sydney, New South Wales Australia; 2 School of Photovoltaic and Renewable Energy Engineering, UNSW Sydney, Sydney, New South Wales Australia; 3 The UNSW RNA Institute, The Australian Centre for Nanomedicine, School of Chemistry, UNSW Sydney, Sydney, New South Wales Australia; 4 Rowland Institute at Harvard University, Cambridge, MA USA; 5 ARC Centre of Excellence in Exciton Science, School of Science, RMIT University, Melbourne, Victoria Australia; 6 Department of Chemistry, School of Physics, Chemistry and Earth Sciences, The University of Adelaide, Adelaide, South Australia Australia

**Keywords:** Excited states, Fluorescence spectroscopy, Light harvesting

## Abstract

The photophysical processes of singlet fission and triplet fusion have numerous emerging applications. They involve the separation of a photo-generated singlet exciton into two dark triplet excitons and the fusion of two dark triplet excitons into an emissive singlet exciton, respectively. The role of the excimer state and the nature of the triplet-pair state in these processes have been a matter of contention. Here we analyse the room temperature time-resolved emission of a neat liquid singlet fission chromophore and show that it exhibits three spectral components: two that correspond to the bright singlet and excimer states and a third component that becomes more prominent during triplet fusion. This spectrum is enhanced by magnetic fields, confirming its origins in the recombination of weakly coupled triplet pairs. It is thus attributed to a strongly coupled triplet pair state. These observations unite the view that there is an emissive intermediate in singlet fission and triplet fusion, distinct from the broad, unstructured excimer emission.

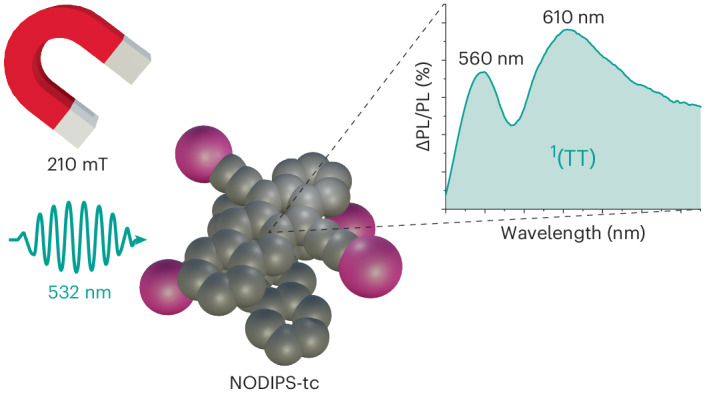

## Abstract (web summary)

Singlet fission produces two molecular excitations from one photon and has the potential to boost solar cell efficiencies. Now it has been shown that magnetic fields can reveal the spectral signatures of the multi-excitonic intermediates in this process.

## Main

Singlet fission (SF)^1^ and triplet fusion (TF)^2^ are related photophysical processes with promising applications spanning photovoltaics^3–5^, photocatalysis^6^, optical and magnetic resonance imaging^7,8^, multi-excitonic logic^9,10^ and advanced manufacturing^11^. In photovoltaics, these processes offer the possibility of much higher limiting efficiencies compared with single-threshold solar cells^3,12–14^. In SF, a photo-prepared excited singlet state couples to a ground-state chromophore and transfers about half its energy, ultimately bringing about two uncoupled chromophores in their lowest-energy triplet states^1^. The system conserves spin and is widely accepted to traverse a region of the potential energy landscape corresponding to a spin-0 (singlet) triplet-pair state, ^1^(TT), with exchange coupling much greater than the zero-field splitting of the individual triplet states^15–17^. As the triplets move apart, the exchange coupling diminishes and the spin-0 character of the bichromophoric state is mixed with other multiplicities^18^. These weakly coupled triplets may return to the singlet manifold by the process of TF^2,18,19^.

In TF, the reverse process occurs whereby weakly coupled triplets become more strongly coupled before crossing to the highly emissive excited singlet state^2^. In concentrated solutions, aggregates and films, the excited singlet state may form an excimer, corresponding to a deep well on the potential energy surface brought about by strong excitonic coupling^20^. The interplay between the excited singlet, S_1_, the excimer, ^1^Ex, and the ^1^(TT) state has been the subject of debate^19,21–28^. In concentrated solutions of 5,12-bis((triisopropylsilyl)ethynyl)tetracene (TIPS-Tc) and 6,13-bis((triisopropylsilyl)ethynyl)pentacene, it has been asserted that the excimer constitutes an intermediate in SF^21,22^. However, though excimers might be involved, Dvořák et al.^23^ showed, using total internal reflection excitation, that concentrated 6,13-bis((triisopropylsilyl)ethynyl)pentacene solutions do not exhibit an emissive excimer. Furthermore, through careful analysis of the time-resolved photoluminescence (TRPL) of concentrated TIPS-Tc solutions, Dover et al.^19^ demonstrated that the excimer, evidenced by a featureless and red-shifted emission spectrum, served as a trap, and that triplets could be generated from the S_1_ state without necessarily accessing the excimeric well. Bossanyi et al.^24^ showed that in pentacene single crystals and 2,8-difluoro-5,11-bis(triethylsilylethynyl)anthradithiophene films at 100 K, a red-shifted spectrum, distinct from the excimer, evinced an emissive intermediate state in TF, which they labelled as ^1^(TT). Such a weakly emissive state was previously observed in low-temperature tetracene thin films^29,30^. The ^1^(TT) intermediate has been the subject of a number of other reports in aggregates and films of acene derivatives^15,31–36^. This begs the question of can such a third, emissive state be observed in SF liquids or solutions? Such an observation would unite models of SF across different phases at device-relevant temperatures. Furthermore, such a spectrum should be enhanced by magnetic fields, which are known to attenuate the generation of free triplets^37,38^.

**Fig. 1:**
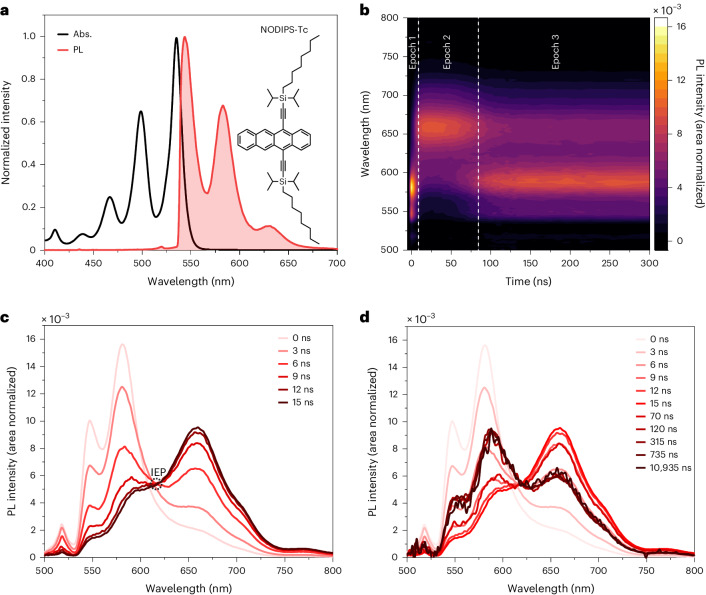
Steady state and TRPL of NODIPS-Tc. **a**, The absorption (Abs.) and fluorescence spectrum of NODIPS-Tc in dilute solution and the chemical structure of NODIPS-Tc. **b**, The normalized-spectrum heat map of TRPL after 532 nm laser excitation. **c**, Area-normalized spectral slices illustrating the early spectral dynamics and isoemissive point (IEP). **d**, Area-normalized spectral slices illustrating the spectral dynamics.

To elucidate this question, we synthesized a room temperature liquid SF material 5,12-bis(*n*-octyldiisopropylsilylethynyl)tetracene (NODIPS-Tc) (Fig. 1a). By adding solvent to the liquid, we bridge between a material composed of neat chromophore, which acts as a solid on experimental timescales, as revealed by molecular dynamics (MD) simulations and solutions of dynamic chromophores. Detailed analysis of the TRPL of this material, both neat and in concentrated solution, reveals three spectral components. The third component, attributed to ^1^(TT), becomes more prominent during TF. Furthermore, the spectrally resolved magnetic field effect (MFE) on the luminescence of this system reveals that luminescence enhancement is explained by enhanced ^1^(TT) emission. This study unites the observations of Dover et al.^19^ and Bossanyi et al.^24^, showing that there is a spectrally observable emissive intermediate in SF both in concentrated solutions and the solid state, but that this is distinct from the broad, red-shifted excimer emission.

## Results

### MD

**Extended Data Fig. 1:**
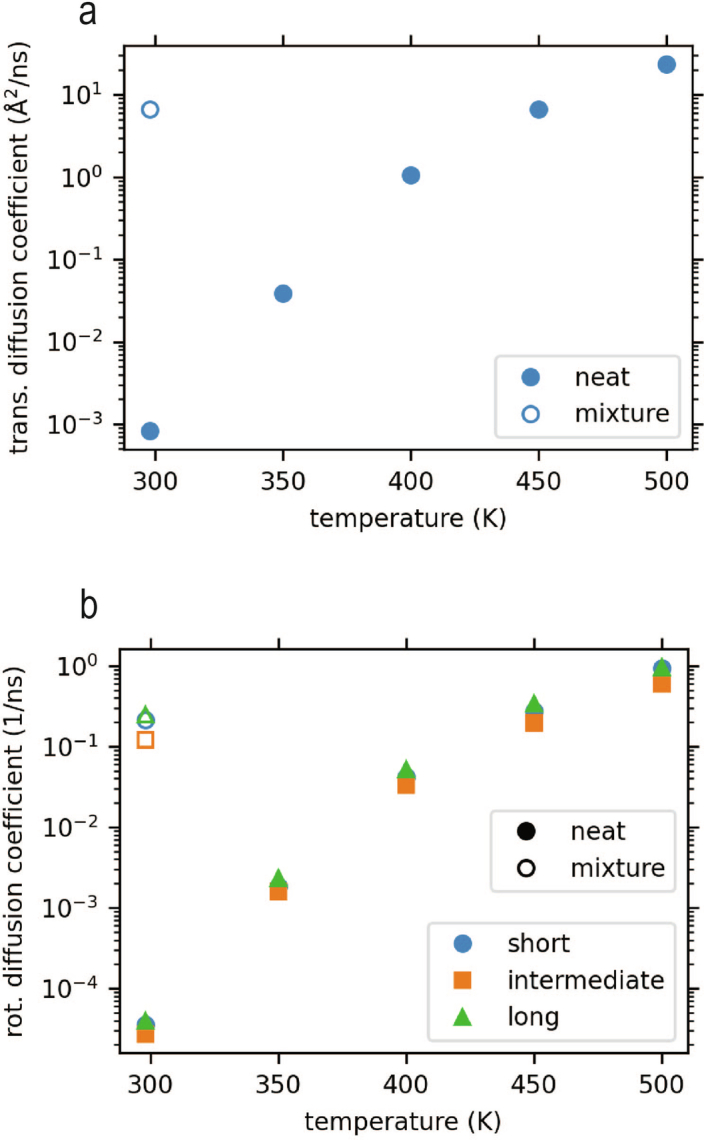
Diffusion coefficients from molecular dynamics. (**a**) Translational diffusion coefficient of the center-of-mass and (**b**) rotational diffusion coefficient of each principal axis (short, intermediate, and long) of the tetracene carbon backbone versus temperature for neat NODIPS-Tc (filled symbols) and 50% w/w NODIPS-Tc/toluene (empty symbols).

MD simulations were performed on neat NODIPS-Tc, and a 50% w/w mixture with toluene. The neat liquid is essentially immobile on experimental timescales, with a structural decorrelation time on the order of 100 μs. However, the structural decorrelation of the mixture was 10^4^ times faster, with a time constant of about 10 ns (Extended Data Fig. 1).

**Extended Data Fig. 2:**
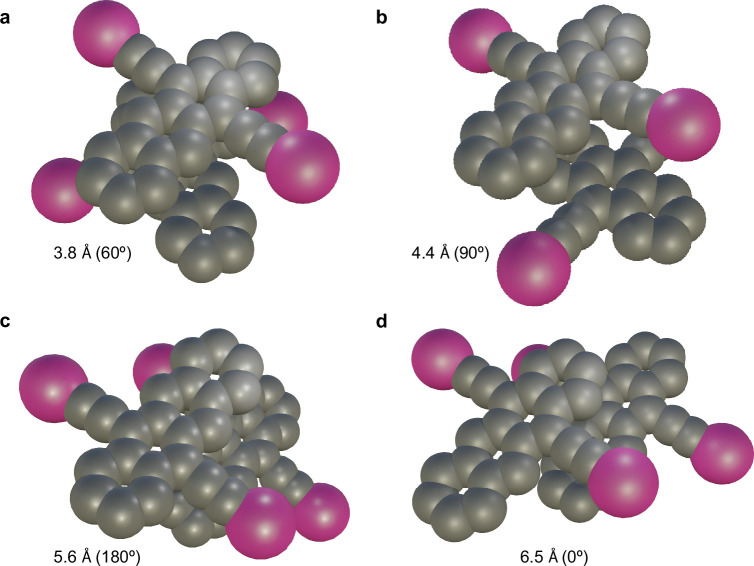
Typical *π*-stacked dimer geometries sampled from the MD simulations corresponding to peaks in the ADRFs. Parameters correspond to centre-of-mass displacement and angle between long-axes. (**a**) 3.8 Å (60°) (**b**) 4.4 Å (90°) (**c**) 5.6 Å (180°) (**d**) 6.5 Å (0°).

The simulations predict that the closest nearest-neighbour interaction of both the neat liquid and the mixture are at a distance of 3.8 Å, with a π-stacking angle of about 60°. The second nearest configuration has the chromophores π-stacked in a perpendicular arrangement at a distance of 4.4 Å (Extended Data Fig. 2). These two arrangements occur as distinct peaks in the configurational probability distributions in the neat liquid, which merge into a single peak/ridge in the mixture (angular–radial distribution functions in Supplementary Figs. 9c and 10c). Configurations expected to be prone to excimer formation, with anti-parallel or parallel transition moments, are found at larger distances of around 5.6 and 6.5 Å, respectively, in both simulations.

### TRPL

**Extended Data Fig. 3:**
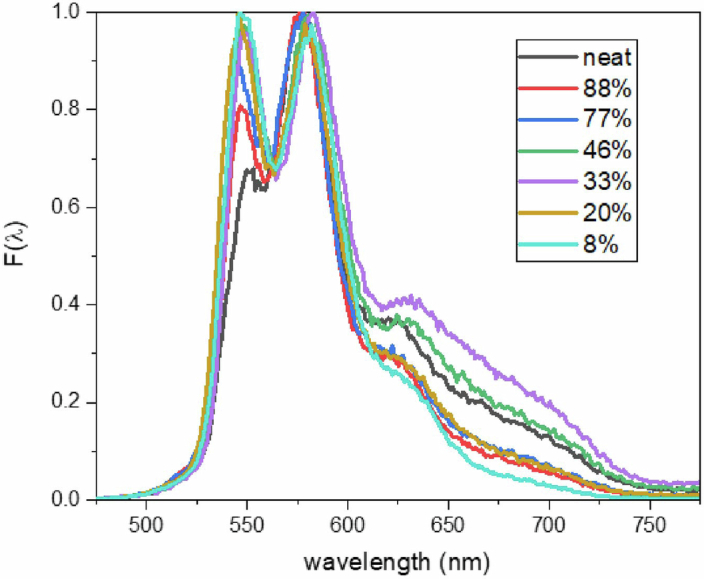
Steady-state photoluminescence spectra of NODIPS-Tc, neat and in toluene at various % w/w concentrations. Samples measured with 532 nm notch filter.

**Extended Data Fig. 4:**
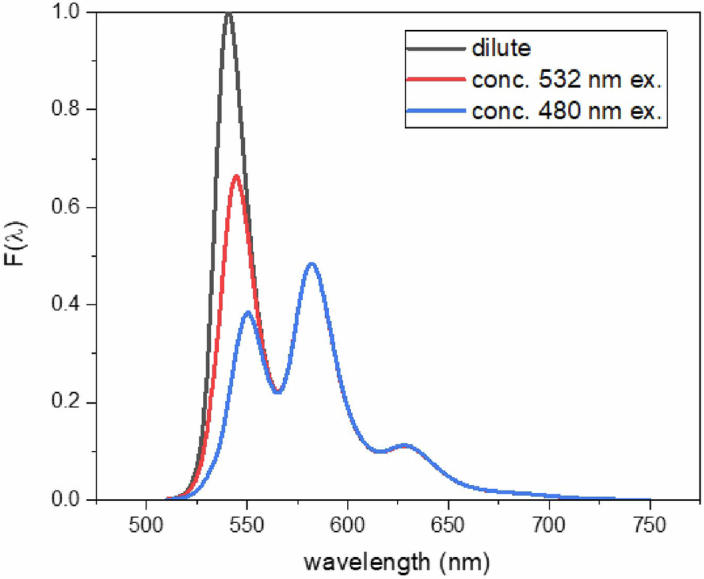
Effect of self-absorption. Steady-state photoluminescence spectrum (black line) of NODIPS-Tc in a solution of toluene at a concentration of 0.01 mg/mL (14 *μ*M) and calculated self-absorbed spectra at excitation wavelengths of 532 nm (red) and 480 nm (blue).

The room temperature TRPL of neat NODIPS-Tc liquid is shown in Fig. 1b. Each spectrum is normalized by its integral, accentuating the changes that occur as the spectrum evolves over time. At early times (0–8 ns, epoch 1), the emission is dominated by the photo-generated S_1_ state, rapidly red shifting into an ^1^Ex-dominated spectral shape in epoch 2 (8–80 ns). The S_1_ emission appears H-aggregated^39^, with the 0–0 band suppressed to a greater extent than in more dilute (yet optically thick) samples measured under identical conditions (Extended Data Fig. 3). This observation is consistent with the predominance of π-stacked nearest neighbours in the molecular dynamics simulations. For comparison, the steady-state absorption and photoluminescence spectra of dilute NODIPS-Tc solution are shown in Fig. 1a. The expected effect of self-absorption on the spectra is shown in Extended Data Fig. 4.

The early time spectral slices spanning epochs 1 and 2 are plotted in Fig. 1c. There is a rapid shift of the iso-emissive point (IEP) in the first few nanoseconds, which then settles down near 620 nm. As with TIPS-Tc, in NODIPS-Tc, both SF and TF pathways are thermally accessible^19,40^. After 100 ns, in epoch 3, the annihilation of SF-generated triplets dominates the spectrum. Spectral slices spanning all epochs are plotted in Fig. 1d.

The presence of a stable IEP after a few nanoseconds in the area-normalized spectra suggests that there are two major spectral components. The IEP deviates in the first few nanoseconds, indicating that there is probably a third component. To quantify the presence of a third component, we implemented principal component analysis (PCA).

### PCA

**Fig. 2:**
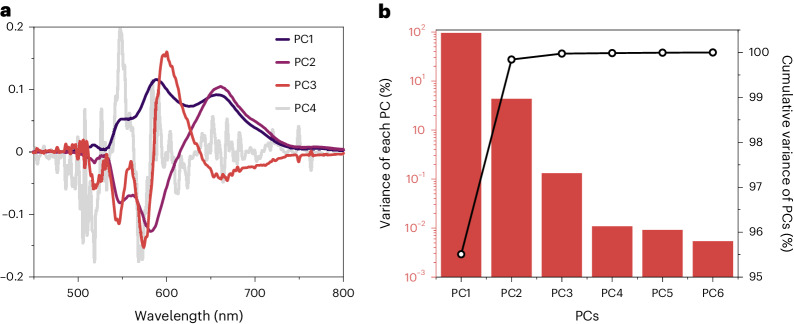
PCA on TRPL of neat NODIPS-Tc. **a**, Eigenvectors of the first four PCs. **b**, Scree plot of variance per cent and cumulative variance per cent of the first six PCs.

**Extended Data Fig. 5:**
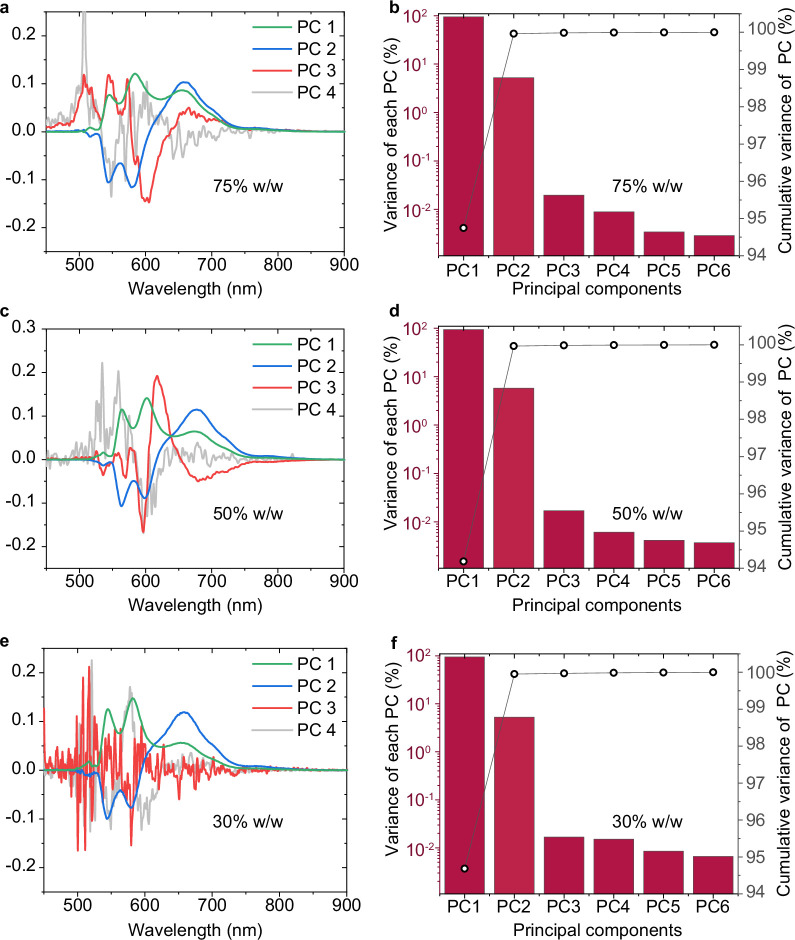
Principal component analysis of the TRPL of dilute solutions. **a** PCs for the 75% w/w sample with PC3 showing signs of being diminished. **b** Scree plot of the 75% w/w sample. **c** PCs of the 50% w/w sample accompanied with **d** a scree plot. **e** PCs of the 30% w/w sample. At this dilution the PC3 is no longer relevant. This is further seen in **f**, the scree plot, which shows a clear elbow at PC3.

The PCA of the dataset is shown in Fig. 2a. A scree plot^41^ showing the percentage variance explained by each principal component (PC) and the cumulative variance is plotted in Fig. 2b. It is important to note that PCA does not generate the spectra of species, and as the PCs are necessarily orthogonal, they can exhibit differently signed spectral regions. PC1 represents the average spectrum and PC2 accounts for the principal spectral changes, with the S_1_ component diminishing and the ^1^Ex component growing in time. The variance attributed to each PC drops steadily before an elbow appears at PC4, indicating the onset of insignificant factors^41^. The eigenvector of PC4 is very noisy. Since three spectra are required to reproduce the deviation from a single IEP in Fig. 1c, and there is no evidence of a significant fourth component, we proceed on the basis that there are three spectral components in the neat sample. The PCA and scree plots for the mixtures with toluene (75%, 50% and 30% w/w) are shown in Extended Data Fig. 5. The third component is not in evidence in these samples as shown by PCA, although the PC3 eigenvector seems to contain spectral information.

The number of PCs may be equated with the number of kinetically and spectrally distinct emissive species. As such, three PCs suggests at least three emissive species. Clearly, during the transition from epoch 1 to 2, the red-shifted ^1^Ex emission grows at the expense of the bluer, S_1_-like spectrum. Since the initially prepared state is S_1_ and the IEP deviates and then settles in the first few nanoseconds, there must be a rapid quasi-equilibrium established between the S_1_ state and another species. We propose that this third spectral component is due to emission by an excitonically coupled chromophore pair, which is spectrally distinct from ^1^Ex. We assign this species to the exchange-coupled triplet pair state, ^1^(TT), as reported in the solid state^24,33,35,36,42,43^.

### Spectral decomposition

**Fig. 3:**
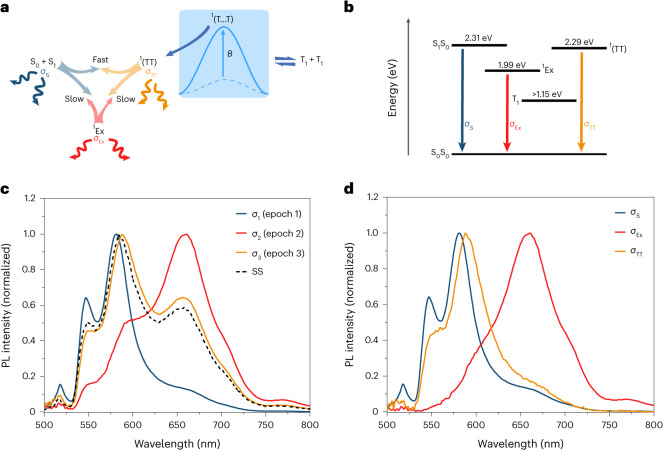
Photoluminescence kinetics. **a**, The kinetic scheme shows the quasi-equilibrium formed between the S_1_ state, ^1^Ex and strongly exchange-coupled ^1^(TT) state, and their connection to separated triplets. The external magnetic field, *B*, modifies the Gibbs free energy of activation for SF, giving rise to the MFE on photoluminescence. **b**, Jablonski diagram of involved states. **c**, Spectra of each epoch and the steady-state (SS) spectrum. **d**, Spectra of each emissive species.

The kinetic scheme is illustrated in Fig. 3a. In epoch 1, there is a rapid onset of equilibrium between S_1_ and ^1^(TT), which then equilibrates with the ^1^Ex state in epoch 2. Dissociation of ^1^(TT) states (SF) generates a pool of free triplets, which then undergo mutual annihilation (TF) in epoch 3.

The spectra of the three epochs, *σ*_1−3_, are shown in Fig. 3c, and are linear combinations of the species-associated spectra, *σ*_S_, *σ*_TT_ and *σ*_Ex_. Epoch 1, *σ*_1_, is clearly *σ*_S_ dominated, and epoch 2 is *σ*_Ex_ dominated. Epoch 3 closely resembles the steady-state emission spectrum, indicating that TF principally populates the S_1_ state after transiting the ^1^(TT) state. Subsequent emission is equivalent to the steady-state spectrum. Since *σ*_3_ is generated by ^1^(TT) states, it will naturally have a higher proportion of ^1^(TT) emission than *σ*_1_ or *σ*_2_.

The spectral evolution that occurs in the first nanoseconds suggests that the ^1^(TT) spectrum, *σ*_TT_, is slightly red shifted compared with the photo-generated S_1_ state spectrum, *σ*_S_, but does not have the same intensity in the deep-red region as the ^1^Ex state spectrum, *σ*_Ex_. As such, we may isolate *σ*_TT_ by assuming that the bluest emission is due to the S_1_ state and the reddest emission is due to the ^1^Ex state. Subtracting contributions due to *σ*_S_, and the excimer-dominated spectrum *σ*_2_, from *σ*_3_ results in the *σ*_TT_ spectrum displayed in Fig. 3d. The other species-associated emission spectra, *σ*_S_ and *σ*_Ex_ are also displayed in Fig. 3d.

### Magnetophotoluminescence

To shed further light on the spectral characteristics of the ^1^(TT) state, we performed magnetic photoluminescence experiments. Though not directly observable through photoluminescence, there must also be a bichromophoric state in which the exchange coupling is weaker than the zero-field splitting, ^1^(T…T). This weakly coupled regime is included in the kinetic scheme illustrated in Fig. 3a. For aligned chromophores, at zero-field there are three out of nine triplet pair sublevels with singlet character ($\left\vert xx\right\rangle ,\left\vert\, yy\right\rangle$ and $\left\vert zz\right\rangle$, where the triplet basis is $\{\left\vert x\right\rangle ,\left\vert\, y\right\rangle ,\left\vert z\right\rangle \}$). As a magnetic field is applied, these states mix with other sublevels and the number of levels with singlet character increases, essentially increasing the entropy of the ^1^(T…T) state. At higher fields where the basis transforms into $\{\left\vert -\right\rangle ,\left\vert 0\right\rangle ,\left\vert +\right\rangle \}$, only two of the nine weakly coupled substates have singlet character, $\left\vert 00\right\rangle$ and $(\left\vert +-\right\rangle +\left\vert -+\right\rangle )/\sqrt{2}$, and the entropy of the state is diminished^37,44–46^. Low entropy of an intermediate is accompanied by a higher free energy of activation, and thus rate constants are effectively manipulated by the magnetic field (Supplementary Information). This effect of the magnetic field on the free energy of the ^1^(T…T) state is illustrated schematically in Fig. 3a.

**Fig. 4:**
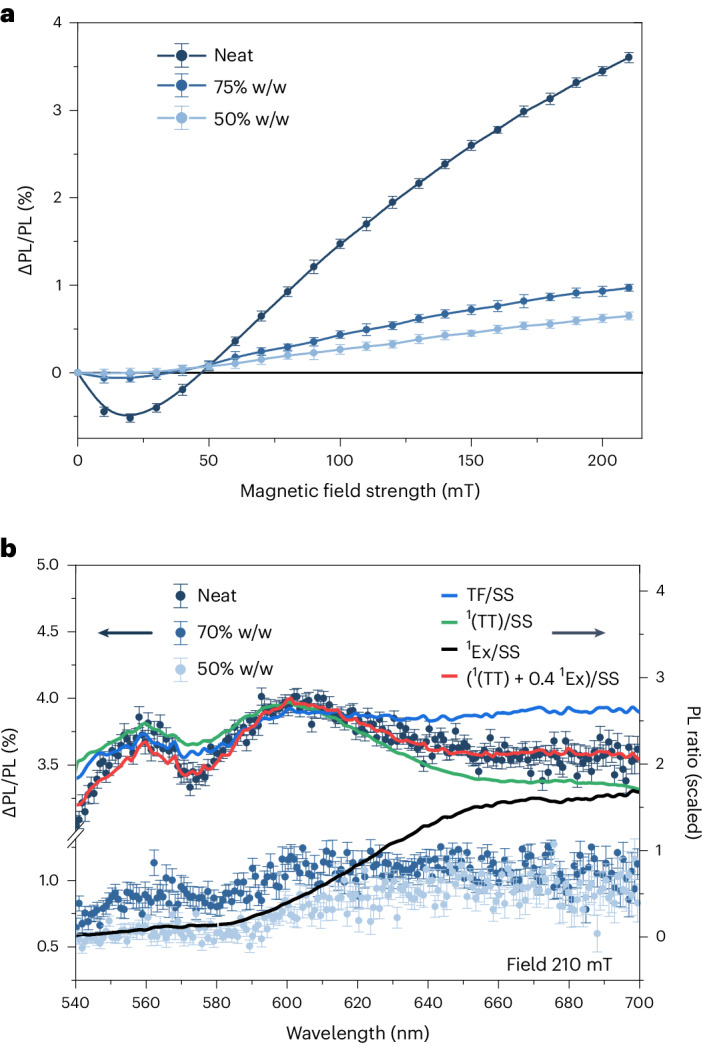
The MFE on photoluminescence of NODIPS-Tc. **a**, The effect of magnetic field (0–210 mT) on the integrated photoluminescence (PL) of NODIPS-Tc at three concentrations. **b**, The wavelength dependence of the MFE on the photoluminescence of NODIPS-Tc at 210 mT magnetic field. The vertical axis is expressed as the percentage photoluminescence change (ΔPL/PL). For comparison, the ratio of the TF spectrum from epoch 3 (*σ*_3_) to the steady-state (SS) spectrum (blue), we plot the ratio of *σ*_TT_ to the steady-state (green), the ratio of the excimer (^1^Ex) to the steady-state and the combination of *σ*_TT_ and *σ*_Ex_ (red). The error bars represent the standard error from 20 individual measurements, errors arise due to small fluctuations in the laser diode power.

The effect of increasing the free energy of the ^1^(T…T) state is to attenuate SF, and thus enhance photoluminescence. In Fig. 4a, we see that at fields as high as 210 mT, for the neat sample, the photoluminescence is enhanced by about 3.6% (ΔPL/PL). Furthermore, we see that at low fields, the photoluminescence is diminished, which is characteristic of chromophores that exhibit hindered rotational diffusion on the timescales of SF and TF^47^. This is less evident but persists in the 75% w/w sample. In the 50% w/w sample, both the effect of hindered rotation and the SF rate are diminished, giving rise to a smaller positive MFE at all fields. This is consistent with the results of the MD simulations, which showed that the mobilities were 10^4^ times higher in the 50% w/w mixture than the neat liquid.

The ΔPL/PL spectrum is shown in Fig. 4b. All samples exhibit peaks around 560 nm and 595 nm. Raising the free energy of the ^1^(T…T) state with a magnetic field should enhance the emission non-uniformly by initially returning a population to the ^1^(TT) state. Indeed, we would expect, as a first approximation, that the magnetic enhancement would be identical to the spectrum observed during epoch 3, where TF dominates. The ratio of the TF spectrum in epoch 3 to the steady-state spectrum is plotted in Fig. 4b (in blue). It exhibits the same shape as the ΔPL/PL spectrum. The population that returned to the S_1_ state by the action of the magnetic field can be expected to have an identical fate to photo-generated S_1_ chromophores, and thus the enhanced emission will be identical to the steady-state emission. Contributions to the ΔPL/PL spectrum from TF-generated S_1_ states will thus result in a featureless ΔPL/PL spectrum. Any spectral features can thus be attributed to excess ^1^Ex or ^1^(TT) states.

The ^1^(TT)-associated spectrum *σ*_TT_ (Fig. 3d) is ratioed to the steady-state spectrum and plotted in Fig. 4b (in green). The ratio of the excimer spectrum to the steady-state spectrum is shown in black. Clearly, the peaks at 560 nm and 595 nm are reproduced by the ^1^(TT) spectrum. However, *σ*_TT_ cannot account for the excess emission in the red region, which must be due to excess excimer formation (compared with the steady-state spectrum). The TF spectrum exhibits a greater excimer contribution than observed in the ΔPL/PL spectrum. The differences suggest that the generation and recombination of geminate ^1^(T…T) pairs does not produce the same spectrum as TF from uncorrelated triplets. It may be hypothesized that the sites that are conducive to SF are those that are less susceptible to excimer formation than a randomly chosen chromophore pair. For instance, the nearest-neighbour sites identified in the MD simulations may act as SF sites that do not easily form excimers due to the hindered mutual rotation of the tetracene subunits. However, the parallel and anti-parallel pairs at longer distance may become engaged in TF through random hopping of triplet excitons and are geometrically predisposed to the formation of an excimer. These dimer geometries are depicted in Extended Data Fig. 2.

**Extended Data Fig. 6:**
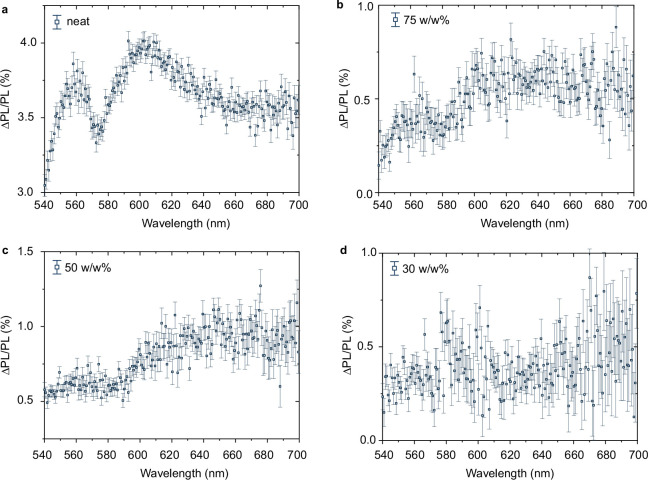
Plots of various concentrations of spectrally resolved MPL at a magnetic field of 210 mT. **a** Shows the neat chromophore, which exhibits peaks at 560 nm and 595 nm. Theses peaks become less pronounced for **b** 75% w/w and **c** 50% w/w. In **d**, for the 30% w/w sample, these peaks are no longer present. The error bars in the plots represent the standard error of the mean after 20 repeat measurements. Error arises due to small fluctuations in the laser diode power.

**Extended Data Fig. 7:**
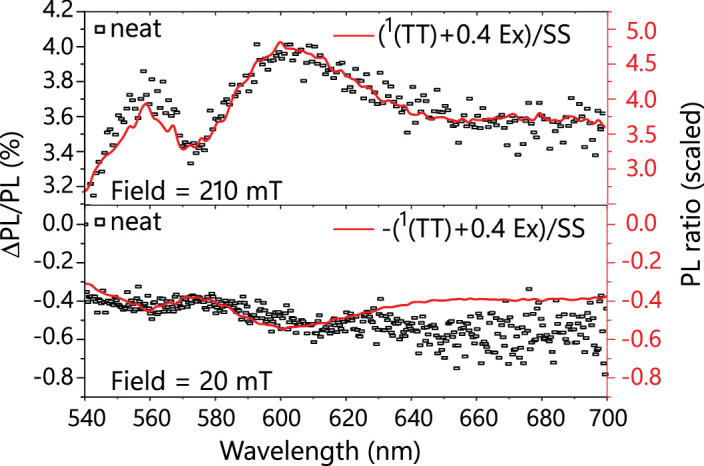
Wavelength dependence of the magnetic-field effect on the PL of NODIPS-Tc at 210 mT and 20 mT magnetic field. The vertical axis is expressed in the percentage PL change (ΔPL/PL). For comparison, we include a best-fit combination of *σ*_TT_ and *σ*_Ex_ ratioed to the steady-state spectrum from the main text (red). The peaks at 560 nm and 595 nm are dips at 20 mT, where the integrated MPL is negative.

MD simulations find that similar dimer geometries occur at 50% w/w in toluene. Figure 4b shows remnants of the spectral features attributed to the ^1^(TT) state, showing that the ^1^(TT) state is in evidence in concentrated solutions, but is displayed to a lesser extent. At 30%, there is no longer any evidence of this emission (Extended Data Fig. 6). Interestingly, at low magnetic fields corresponding to the dip in Fig. 4a, a spectrum showing negative ^1^(TT) features is observed (Extended Data Fig. 7).

**Extended Data Fig. 8:**
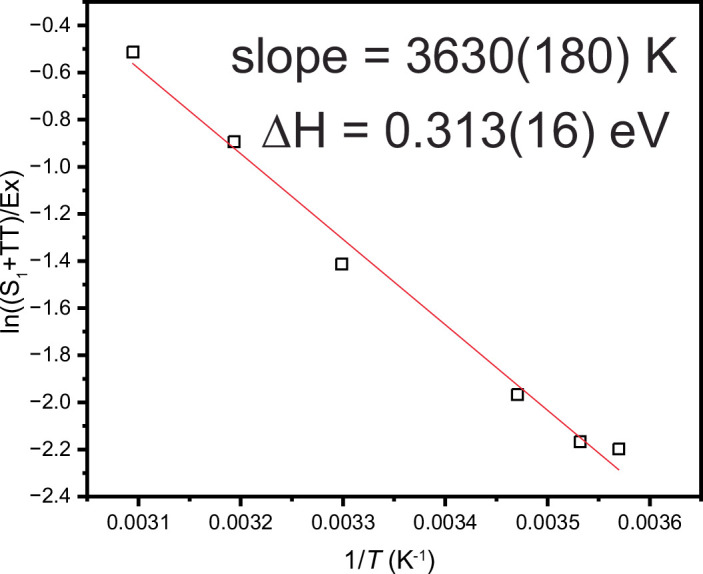
Plot of the logarithm of the ratio of the sum of S_1_ and ^1^(TT) components to the ^1^Ex component against inverse temperature for the spectra of Epoch 2. The linear fit indicates that the excimer is 0.31 eV below the other singlet states. The intercept is not meaningful without information on the relative radiative rates of these states.

The shape of *σ*_TT_ is essentially that of a broadened and red-shifted S_1_ spectrum. The broadening can be attributed to a distribution of chromophore pair geometries, as predicted by the MD simulations (Supplementary Figs. 9–11). The energy of the ^1^(TT) state is estimated to be about 2.30 eV. Notwithstanding the triplet binding energy, this places the *T*_1_ state of the NODIPS-Tc chromophore at >1.15 eV, consistent with energy transfer experiments^48^. The energy of the excimer state was estimated from a van’t Hoff plot, shown in Extended Data Fig. 8. The S_1_ and ^1^(TT) emission is activated by 0.313(16) eV, placing the ^1^Ex state at 1.99 eV. The relative energies of these states are shown in Fig. 3b.

The results in this work underscore the complexity of the excited states in the most studied SF chromophore (tetracene). In addition to unifying the debate regarding the fundamental photophysics of SF and TF in solids and solutions, our results have many implications for technologies that exploit SF and TF. First, multi-exciton logic and magnetic resonance imaging are underpinned by an understanding of spin evolution and is affected by the equilibrium in Fig. 3a. Second, the development of TF-based light-emitting diodes necessitates an understanding of all the emissive states in the system. Finally, magnetic field dependent measurements are often used to characterize SF-based solar cells^5,49,50^. These analyses usually assume that the magnetic field perturbs the equilibrium between (S_0_S_1_ and T_1_ + T_1_), mediated by the singlet character of the ^1^(T…T) pair. However, as shown in Fig. 4, this is an oversimplication, as it does not consider the interplay between these states and the coupled-pair ^1^(TT) and ^1^Ex states.

## Conclusions

In this study, we analysed the time-resolved emission of NODIPS-tetracene, a liquid-state SF and TF material. As seen in other systems, the photo-generated bright state generates excimers that emit with an unstructured, red-shifted spectrum. In the first few nanoseconds, there is a shift of the IEP, which settles down near 620 nm and remains in place at longer times, during the TF phase. This initial shift in the IEP indicates the presence of a third spectral component, which is confirmed by PCA.

Further insight into the spectral components generated by a triplet-pair is gained from magnetic field experiments. Photoluminescence enhancement is observed at particular wavelengths, as well as in the excimer spectrum, compared with the steady-state spectrum. The peaks in the ΔPL/PL spectrum are assigned to the ^1^(TT) state. MD simulations show that the neat chromophore behaves essentially as an amorphous solid on experimental timescales, but that the 50% mixture with toluene is dynamic. They both exhibit the same features in the magnetic luminescence attributed to the ^1^(TT) state, demonstrating a common SF mechanism in two very different states of matter.

These results highlight several features of SF and TF systems in general. They further show that the excimer state is not a necessary intermediate in SF or TF, but serves as a trap. Furthermore, it is shown that emission from the ^1^(TT) state can be observed at room temperature in non-solid state systems, and that this emission is enhanced by a magnetic field more so than the steady-state emission. In more dilute solutions of chromophore, these effects are less evident.

## Methods

### MD

MD simulations were carried out using the Large-scale Atomic/Molecular Massively Parallel Simulator^51–53^ (2 August 2023 version with graphic processing unit acceleration). Short-ranged non-bonded interactions were cut off at a distance of 11 Å, while the long-ranged part of electrostatic interactions were computed with the particle–particle particle–mesh method^53,54^. Bond lengths involving hydrogen atoms were constrained using the SHAKE algorithm^55^. Constant temperature and pressure were maintained using a Nosé–Hoover^56,57^ style thermostat and barostat^58^ in a cubic simulation box with periodic boundary conditions enforced in all dimensions.

Two systems were simulated: a pure NODIPS-Tc system containing 216 molecules and an approximately 50% w/w NODIPS-Tc/toluene mixture containing 108 NODIPS-Tc molecules and 855 toluene molecules. For each system, the initial configuration was built using Moltemplate^59^ (version 2.20.19), with molecules placed on a cubic grid at very low density to avoid overlapping molecules. The system energy was minimized and then a short (50–75 ps) simulation was carried out at constant volume and temperature at a a high temperature of 600 K, during which the simulation timestep was progressively increased to its final value. The simulation was then continued at constant pressure and temperature at 600 K and 100 atm for at least 1 ns to shrink the system to liquid density and to randomize the molecular positions and orientations. For the NODIPS-Tc/toluene mixture, the system was then simulated in the ensemble at 298 K and 1 atm. Owing to the low molecular mobility of pure NODIPS-Tc at room temperature, equilibration of this system at 1 atm was carried out in stages, with the temperature progressively decreased from 500 K to 450 K to 400 K to 350 K to 298 K, with sufficient time spent at each intermediate temperature to reach equilibrium. In addition, between the 350 K and 298 K simulations, the temperature was decreased at a constant rate over a period of 150 ns. Details of the simulations that were performed are given in Supplementary Table 6.

### Chemical synthesis

NODIPS acetylene was prepared as previously reported^60^. See Supplementary Information for full details of the synthetic procedures^60–62^.

### Sample preparation

All samples used for TRPL spectroscopy were measured under an inert atmosphere using a Teflon-stopcock sealed quartz cuvette with a 1 mm path length. Toluene was added for diluted samples.

### Optical spectroscopy

TRPL was carried out using a SpitLight Picolo, ND:YVO_4_ laser system with pulse duration of 800 ps at a repetition rate of 1 kHz and wavelength of 532 nm. Photoluminescence was detected using a spectrometer (Princeton Instruments 2300i) equipped with a 300 groove-per-millimetre grating blazed at 500 nm, and an intensified time-gated camera (Princeton Instruments Pi Max-4).

Steady-state optical absorption spectroscopy was performed with a Cary 60 ultraviolet–visible–near infrared spectrometer covering a wavelength range of 190–1,100 nm.

For steady-state photoluminescence measurements, samples were excited by a 532 nm continuous-wave laser. The photoluminescence was collected and imaged onto a multi-mode optical fibre by two off-axis parabolic mirrors. An Ocean Optics Flame spectrometer was used to collect the photoluminescence spectrum. The magnetic field was provided by a benchtop electromagnet (Magnetech MFG-6-24) controlled by a d.c. power supply (Keithley 2230G-30-1).

### PCA

PCA was used to extract the emissive species (PCs) contributing to the TRPL. These PCs form a new basis set and can be used to reconstruct the experimental data. First, the experimental TRPL dataset was organized as an *m* × *n* matrix *X*, where *m* is the number of measurement types, that is, dimensions (in this case the number of wavelengths) and *n* is the number of observations (in this case the number of time series). Second, the covariance matrix *C* = *XX*^T^ was computed, where *C* is a square symmetric *m* × *m* matrix. The diagonal terms of *C* are the variance of each measurement type and the off-diagonal terms are the covariance between measurement types. The *C* matrix contains the correlations between all possible pairs of measurements. Last, eigendecomposition of the covariance matrix *C* was performed, which yields the eigenvectors of PCs and eigenvalues, that is, the variance of each PC.

## Online content

Any methods, additional references, Nature Portfolio reporting summaries, source data, extended data, supplementary information, acknowledgements, peer review information; details of author contributions and competing interests; and statements of data and code availability are available at 10.1038/s41557-024-01591-0.

## Supplementary information

Supplementary Information Supplementary Text Sections 1–7, Figs. 1–19, Tables 1–6 and references.

## Data Availability

Data supporting Figs 1–4 are publicly available at 10.5061/dryad.05qfttf8r.
