# Supplementary material for: Magnetic fields reveal signatures of triplet-pair multi-exciton photoluminescence in singlet fission
Source: Nat Chem. 2024 Jul 25;16(11):1861–7. doi: 10.1038/s41557-024-01591-0 (PMC11527793; doi:10.1038/s41557-024-01591-0)
Supplement: Supplementary file 1 — Supplementary Text Sections 1–7, Figs. 1–19, Tables 1–6 and references. [file 41557_2024_1591_MOESM1_ESM.pdf]

nature chemistry

Supplementary information https://doi.org/10.1038/s41557-024-01591-0

Magnetic fields reveal signatures of triplet-pair multi-exciton photoluminescence in singlet fission

In the format provided by the authors and unedited

Contents

1 Steady-state spectroscopy **2**

2 Time-resolved photoluminescence of toluene mixtures **4**

3 Entropy of the $^1(\mathrm{T}\ldots\mathrm{T})$ intermediate **5**

4 Molecular dynamics (MD) simulations **7**
4.1 Simulation parameters 7
4.2 Simulation method 12
4.3 Simulation results 13
4.3.1 Dynamics 13
4.3.2 Structure 17

5 Detailed synthetic procedure **22**
5.1 Synthesis of n-octyl(diisopropyl)silylacetylene (NODIPS acetylene) 22
5.2 Synthesis of 5,12-bis((diisopropyl(octyl)silyl)ethynyl)tetracene (NODIPS-Tc) 22

6 Integrated Magnetic Photoluminescence **28**

7 van 't Hoff Plot **29**

1 Steady-state spectroscopy

In optically thick samples, the excitation beam propagates into the sample and is absorbed according to the Beer-Lambert Law, with coefficient α_{ex}, generating a normalized concentration profile

$$n(z) = \alpha_{\text{ex}} \exp(-\alpha_{\text{ex}} z) \tag{1}$$

When photoluminescence is emitted back towards the excitation, the emitted light is absorbed with coefficient α_λ, and the observed emission intensity is an integral over the spatial excitation profile

$$\begin{aligned} F(\lambda) &= F_0(\lambda) \int_0^\infty \alpha_{\text{ex}} \exp(-\alpha_{\text{ex}} z) \exp(-\alpha_\lambda z) dz \\ &= \frac{\alpha_{\text{ex}}}{\alpha_{\text{ex}} + \alpha_\lambda} F_0(\lambda) \end{aligned} \tag{2}$$

This results in the suppression of the 0–0 band. A calculated spectrum for excitation at 532 nm is shown below in Supplementary Fig. 1.

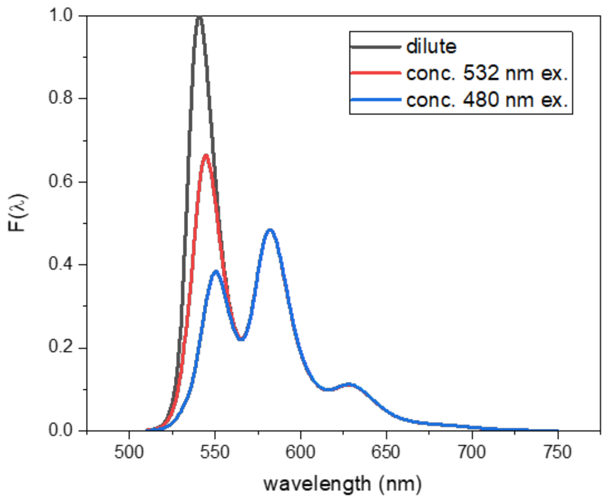

Supplementary Figure 1: Steady-state photoluminescence spectrum (black line) of NODIPS-Tc in a solution of toluene at a concentration of 0.01 mg/mL (14 μM) and calculated self-absorbed spectra at excitation wavelengths of 532 nm (red) and 480 nm (blue).

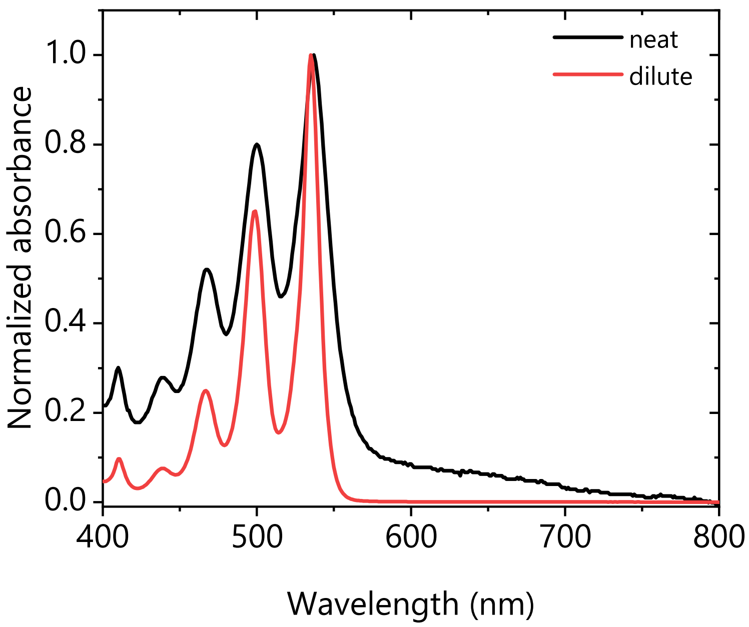

Supplementary Figure 2: Steady-state absorbance spectrum (red line) of NODIPS-Tc in a solution of toluene at a concentration of 0.01 mg/mL and a neat absorbance spectrum (black line).

2 Time-resolved photoluminescence of toluene mixtures

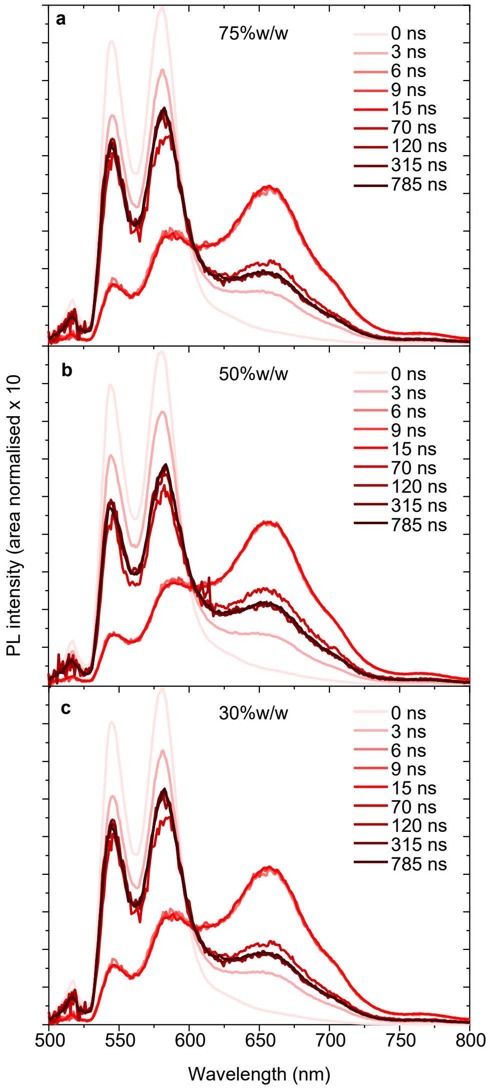

Supplementary Figure 3: Time slices from area normalised TRPL spectra of **(a)** 75% w/w, **(b)** 50% w/w, and **(c)** 30% w/w of NODIPS-Tc in toluene.

3 Entropy of the $^1(\mathrm{T}\ldots\mathrm{T})$ intermediate

We consider the rate of singlet fission from some strongly exchange-coupled state S_1 (which includes $^1(\mathrm{TT})$) to uncoupled triplet pairs (T + T) via weakly coupled pairs ($^1(\mathrm{T}\ldots\mathrm{T})$). The latter is treated as an intermediate.

$$\mathrm{S}_1 \underset{k_{-1}}{\overset{k_1}{\leftrightharpoons}} (\mathrm{TT}) \overset{k_2}{\rightarrow} \mathrm{T}+\mathrm{T} \tag{3}$$

The first reaction is reversible, but we only consider the forward direction of the second here. Now, assuming a steady state concentration of (TT), we note that for the ith pair with c_i singlet character ($\sum_i c_i = 1$),

$$[(\mathrm{TT})_i](c_i k_{-1} + k_2) = c_i k_1 [\mathrm{S}_1] \tag{4}$$

Thus,

$$[(\mathrm{TT})_i] = \frac{c_i k_1 [\mathrm{S}_1]}{c_i k_{-1} + k_2}$$
$$K_i^\ddagger = \frac{[(\mathrm{TT})_i]}{[\mathrm{S}_1]} = \frac{c_i k_1}{c_i k_{-1} + k_2} \tag{5}$$

Now, the total rate is

$$\text{rate} = k_{\mathrm{SF}}[\mathrm{S}_1] = \sum_i k_2 \frac{c_i k_1 [\mathrm{S}_1]}{c_i k_{-1} + k_2} = \sum_i k_2 K_i^\ddagger [\mathrm{S}_1] = k_2 K^\ddagger [\mathrm{S}_1] \tag{6}$$

In the spirit of Eyring [1], we equate the quasi-equilibrium constant $K^\ddagger$ with the exponentiated free energy of activation,

$$K^\ddagger = \exp\left(-\frac{\Delta G^\ddagger}{RT}\right) \tag{7}$$

and as such,

$$\Delta G^\ddagger = -RT \ln\left(\sum_i \frac{c_i k_1}{c_i k_{-1} + k_2}\right) \tag{8}$$

In the case that the singlet character is equally distributed over W states,

$$\Delta G^\ddagger = -RT \ln\left(\frac{k_1}{k_{-1}/W + k_2}\right) \tag{9}$$

which decreases with increasing W. We suppose that the action of a magnetic field has very little effect on the enthalpy. Therefore, we can attribute the spread of the singlet character to an entropy of activation which we reference for convenience to $W = 1$.

$$\Delta\Delta S^\ddagger(\{c_i\}) = R \ln\left(\sum_i \frac{c_i k_{-1} + c_i k_2}{c_i k_{-1} + k_2}\right) \tag{10}$$

In the case that the singlet character is distributed over W states,

$$\Delta\Delta S^{\ddagger}(W) = R\ln\left(\frac{k_{-1}+k_2}{k_{-1}/W+k_2}\right) \tag{11}$$

which is monotonically increasing in W. Where $W=1$, this quantity is zero, as expected. If $k_2 \gg k_{-1}$, no effect is seen. If $k_2 = k_{-1}$, then

$$\Delta\Delta S^{\ddagger}(W) = R\ln\left(\frac{2W}{W+1}\right) \tag{12}$$

As k_2 becomes small, then

$$\Delta\Delta S^{\ddagger}(W) = R\ln(W) \tag{13}$$

4 Molecular dynamics (MD) simulations

4.1 Simulation parameters

The Optimized Potentials for Liquid Simulations–All Atom (OPLS-AA) force field [2–4] as implemented in Moltemplate (version 2.20.19) [5] was used in molecular dynamics (MD) simulations of NODIPS-Tc and NODIPS-Tc/toluene mixtures. The atom and bond atom types in the NODIPS-Tc and toluene models are defined in Supplementary Fig. 4 and the simulation parameters are given in Supplementary Tables 1–5. Where parameters were not defined in the force field, which was the case for bonded interactions involving the silylethynl moiety, parameters for the most closely analogous interactions defined in the force field were used, as indicated in Supplementary Tables 2–4. The implementation of improper dihedral potentials in Moltemplate was also modified to more accurately match that in the OPLS-AA force field, as described in Supplementary Table 5.

Supplementary Table 1: Masses, Lennard-Jones (LJ) parameters, and partial charges of atom types. The non-bonded potential between atoms of type i and j separated by distance r_{ij} is $U_{\mathrm{nb}} = 4\epsilon_{ij}\left[\left(\frac{\sigma_{ij}}{r_{ij}}\right)^{12} - \left(\frac{\sigma_{ij}}{r_{ij}}\right)^{6}\right] + \frac{q_i q_j}{4\pi\epsilon_0 r_{ij}}$, where σ_i is the homonuclear LJ diameter, ϵ_i the homonuclear LJ interaction strength, q_i the partial charge, and heteronuclear parameters were derived from homonuclear ones using geometric mixing rules, i.e. $\sigma_{ij} = \sqrt{\sigma_i \sigma_j}$ and $\epsilon_{ij} = \sqrt{\epsilon_i \epsilon_j}$. 1–2 and 1–3 interactions (between atoms separated by one and two bonds, respectively) were set to zero, and 1–4 interactions (between atoms separated by three bonds) were set to 0.5 times their full values.

atom type	mass (g/mol)	ϵ_i (kcal/mol)	σ_i (Å)	q_i (e)
80	12.011	0.066	3.5	-0.18
81	12.011	0.066	3.5	-0.12
85	1.008	0.03	2.5	0.06
90	12.011	0.07	3.55	-0.115
91	1.008	0.03	2.42	0.115
92	12.011	0.07	3.55	0.0
93	12.011	0.066	3.5	-0.065
769	12.011	0.21	3.3	0.0
769*[1]	12.011	0.21	3.3	-0.25
866	28.086	0.1	4.0	1.0
872	12.011	0.066	3.5	-0.37
873	12.011	0.066	3.5	-0.31

[1] Atom type identical to 769 except that its partial charge has been adjusted to enforce overall charge neutrality of the NODIPS-Tc molecule.

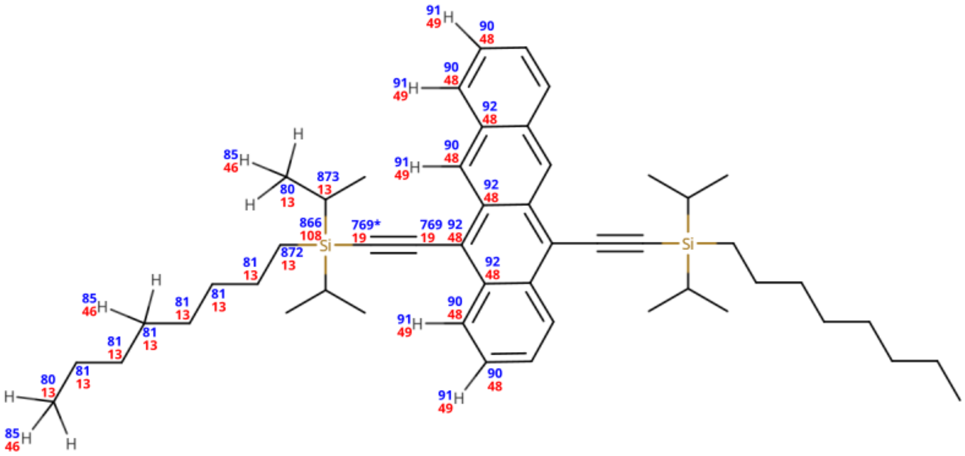

(a) NODIPS-Tc

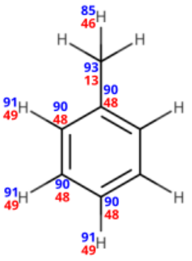

(b) toluene

Supplementary Figure 4: Definition of atom types (blue) and bond atom types (red) for the parameterisation of (a) NODIPS-Tc and (b) toluene with the OPLS-AA force field. For clarity, only some hydrogen atoms are depicted explicitly in NODIPS-Tc and atoms are not numbered if there is an equivalent numbered atom in the molecule. The atom and bond type numbering follows the convention used in the `oplsaa.prm` file distributed with Tinker [6], which is in turn followed in the `oplsaa.lt` file distributed with Moltemplate [5]. Atom type 769* is identical to OPLS-AA atom type 769 except that its partial charge has been adjusted to enforce overall charge neutrality of the NODIPS-Tc molecule (see Supplementary Table 1).

Supplementary Table 2: Parameters in bond-length potential $U_{\text{bond}}(l) = k_{\text{b}}(l - l_0)^2$, where l is the bond length and l_0 is the equilibrium bond length.

bond type	k_{b} (kcal/mol/Å^2)	l_0 (Å)
13–13	268	1.529
13–46	340	1.09
13–48	317	1.51
13–108	187	1.86
19–19	1150	1.21
19–48	400	1.451
19–108[1]	187	1.86
48–48	469	1.4
48–49	367	1.08

[1]Not defined in OPLS-AA. Parameters for 13–108 bond type used.

Supplementary Table 3: Parameters in angle-bending potential $U_{\text{angle}}(\theta) = k_{\text{a}}(\theta - \theta_0)^2$, where θ is the bond angle and θ_0 is the equilibrium angle.

angle type	k_{a} (kcal/mol/rad^2)	θ_0 (degree)
13–13–13	58.35	112.7
13–13–46	37.5	110.7
13–13–108	60	112
46–13–46	33	107.8
46–13–48	35	109.5
46–13–108	35	109.5
19–19–48	160	180
19–19–108[1]	150	180
13–48–48	70	120
19–48–48	70	120
48–48–48	63	120
48–48–49	35	120
13–108–13	60	110
13–108–19[2]	60	110

[1]Not defined in OPLS-AA. Parameters for 13–19–19 angle type used.

[2]Not defined in OPLS-AA. Parameters for 13–108–13 angle type used.

Supplementary Table 4: Parameters in proper dihedral potential $U_{\mathrm{dihedral}}(\phi) = \frac{1}{2}K_1\left[1+\cos(\phi)\right] + \frac{1}{2}K_2\left[1-\cos(2\phi)\right] + \frac{1}{2}K_3\left[1+\cos(3\phi)\right] + \frac{1}{2}K_4\left[1-\cos(4\phi)\right]$, where ϕ is the dihedral angle. "X" is a wild-card atom.

dihedral type	K_1 (kcal/mol)	K_2 (kcal/mol)	K_3 (kcal/mol)	K_4 (kcal/mol)
13–13–13–13	1.3	-0.05	0.2	0
13–13–13–46	0	0	0.3	0
13–13–13–108	0	0	0	0
46–13–13–46	0	0	0.3	0
46–13–13–108	0	0	0.45	0
46–13–48–48	0	0	0	0
13–13–108–13	1	0	0	0
13–13–108–19[1]	1	0	0	0
46–13–108–13	0	0	0.18	0
46–13–108–19[2]	0	0	0.18	0
X–19–19–X	0	0	0	0
19–19–48–48[3]	0	0	0	0
19–19–108–13[3]	0	0	0	0
13–48–48–49	0	7.25	0	0
48–48–48–48	0	7.25	0	0
48–48–48–49	0	7.25	0	0
49–48–48–49	0	7.25	0	0
X–48–48–48	0	7.25	0	0

[1] Not defined in OPLS-AA. Parameters for 13–13–108–13 diheral type used.

[2] Not defined in OPLS-AA. Parameters for 46–13–108–13 dihedral type used.

[3] Not defined in OPLS-AA. Parameters for 13–13–19–19 dihedral type used.

Supplementary Table 5: Parameters in improper dihedral potential $U_{\mathrm{improper}}(\chi) = K[1 + d\cos(n\chi)]$,[1] where χ is the improper dihedral angle. “X" is a wild-card atom.

improper type	K (kcal/mol)	d	n
X–X–48–X[2]	1.1	-1	2

[1]This form of the potential corresponds to that used in the OPLS-AA forc field, while Moltemplate [5] uses a potential of the form $U_{\mathrm{improper}}(\chi) = K(\chi - \chi_0)^2$, where χ_0 is the equilibrium improper dihedral angle.

[2]The OPLS-AA force field applies the improper dihedral potential only to one of the improper dihedrals that can be defined for a given central atom. To avoid ambiguity in the choice of this angle, we have distributed this potential evenly over the three non-redundant improper dihedrals that can be defined for a given central atom bonded to three other atoms, i.e. by applying the potential to each angle with amplitude $K/3$.

4.2 Simulation method

See Methods section of main text.

Supplementary Table 6: Details of simulated systems: $N_{\mathrm{NODIPS-Tc}}$ = number of NODIPS-Tc molecules, N_{toluene} = number of toluene molecules, T = temperature, Δt = simulation time step, t_{tot} = total simulation time, t_{equil} = equilibration time (initial simulation time not used in measurements of observables), $\langle V \rangle$ = average system volume, $\langle \rho \rangle$ = average density (pressure = 1 atm in all simulations).

$N_{\mathrm{NODIPS-Tc}}$	N_{toluene}	T (K)	Δt (fs)	t_{tot} (ns)	t_{equil} (ns)	$\langle V \rangle$ (nm^3)	$\langle \rho \rangle$ (g/cm^3)
216	0	500	1	10	2.5	$(6.91)^3$	0.793
216	0	450	1	10	2.5	$(6.79)^3$	0.834
216	0	400	1	30	6	$(6.69)^3$	0.873
216	0	350	1.5	180	45	$(6.59)^3$	0.914
216	0	298	1.5	180	45	$(6.51)^3$	0.948
108	855	298	1	20	5	$(6.64)^3$	0.893

4.3 Simulation results

4.3.1 Dynamics

The mean squared displacement (MSD), $\Delta\mathbf{r}^2(t) = \left\langle|\mathbf{r}(t+\tau) - \mathbf{r}(\tau)|^2\right\rangle$, as a function of time t of the center-of-mass position $\mathbf{r}$ of the tetracene carbon backbone of NODIPS-Tc was calculated in each simulation by averaging over all the molecules and over the initial time τ. The MSD is plotted versus time for all the simulations in Supplementary Figure 5. The translational diffusion coefficient D_T was calculated as 1/6 of the slope of the curve at long times and is plotted in Supplementary Figure 7a as a function of temperature for neat NODIPS-Tc as well as at 298 K for the NODIPS-Tc/toluene mixture.

The orientational time correlation function (OTCF), $F_i(t) = \langle\hat{\mathbf{u}}_i(t+\tau)\cdot\hat{\mathbf{u}}_i(\tau)\rangle$, as a function of time t of the unit vector $\hat{\mathbf{u}}_i$ aligned along each principal axis i of the tetracene carbon backbone of NODIPS-Tc was calculated in each simulation by averaging over all the molecules and over the initial time τ. The principal axes of a molecule were calculated as the eigenvectors of the inertia tensor of the tetracene carbon backbone in each simulation configuration, with the short a-axis normal to the tetracene plane, the intermediate b-axis in the plane and perpendicular to the tetracene backbone, and the long c-axis in the plane and aligned with the backbone. The OTCF is plotted versus time for each principal axis for all the simulations in Supplementary Figure 6. The rotational diffusion coefficient $D_{\mathrm{R},i}$ was calculated for each axis i by fitting the OTCF to an exponential with decay constant $2D_{\mathrm{R},i}$ and is plotted in Supplementary Figure 7b as a function of temperature for neat NODIPS-Tc as well as at 298 K for the NODIPS-Tc/toluene mixture.

Note that both the translational and rotational diffusion coefficients measured in MD simulations depend on the simulation box size [7, 8] and in principle should be corrected for the finite system size. Using the box length in our simulations of roughly 6–7 nm and assuming a hydrodynamic radius of a NODIPS-Tc molecule of approximately 1–1.5 nm, the finite-size corrections to the translational and rotational diffusion coefficients are estimated to be less than 10% and 5%, respectively, so the values in Supplementary Figure 7 have not been corrected.

The translational and rotational diffusion coefficients of neat NODIPS-Tc at 298 K in Supplementary Figure 7 are on the order of 10^{-3} Å^2/ns and 10^{-5}/ns, respectively, which means that the system is essentially immobile on the $\sim$100 ns time scale of the simulation. Estimating the time scale of structural decorrelation to be the time required for a NODIPS-Tc molecule to diffuse the length of the tetracene backbone of $\approx$ 10 Å ($\Delta\mathbf{r}^2(t) \approx 100$ Å^2) or as the inverse of the rotational diffusion coefficient gives a decorrelation time on the order of 100 microseconds. By contrast, the corresponding diffusion coefficients of the NODIPS-Tc/toluene mixture at 298 K are on the order of 10^4 times as large, with structural decorrelation occurring on the 10 ns time scale.

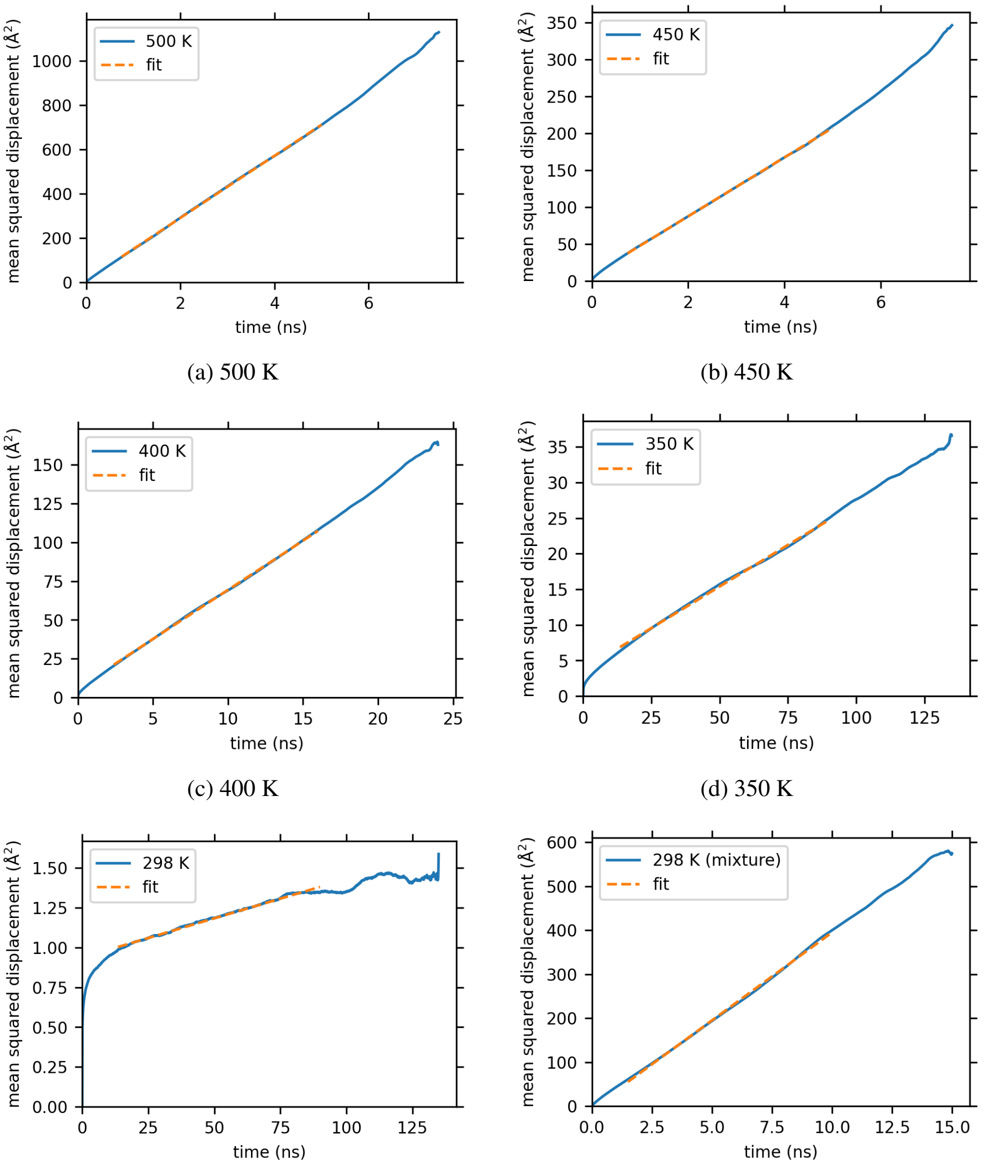

(a) 500 K
(b) 450 K
(c) 400 K
(d) 350 K
(e) 298 K
(f) 298 K (mixture)

Supplementary Figure 5: Mean squared displacement (MSD) versus time (solid line) of the center-of-mass of the tetracene carbon backbone of neat NODIPS-Tc at (a) 500 K, (b) 450 K, (c) 400 K, (d) 350 K, and (e) 298 K, and of (f) 50% w/w NODIPS-Tc/toluene at 298 K. The dashed line is a linear fit to the data that excludes the first tenth and last third of the data to limit fitting to the linear region, from which the translational diffusion coefficient D_{T} was obtained as 1/6 of the slope.

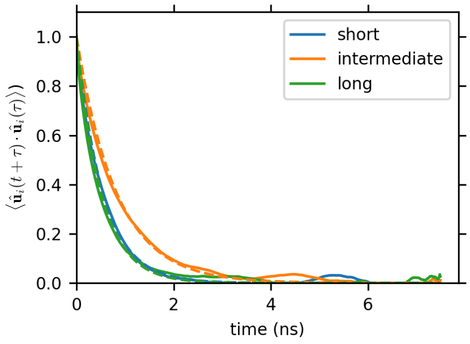

(a) 500 K

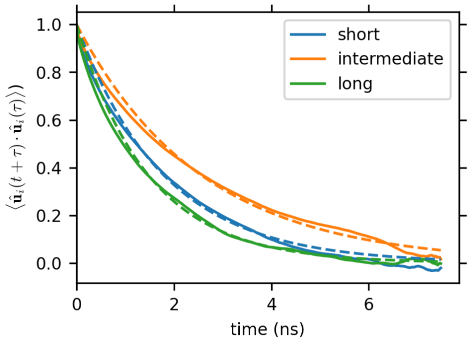

(b) 450 K

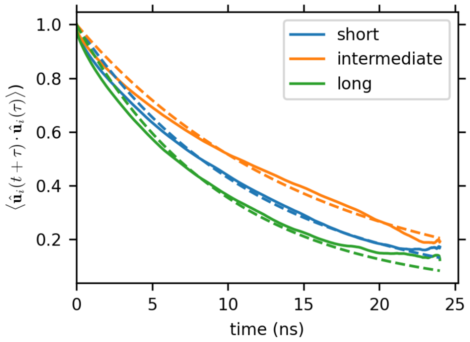

(c) 400 K

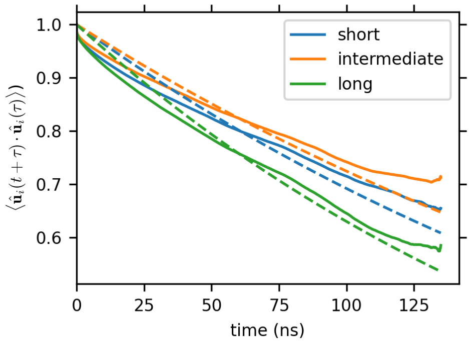

(d) 350 K

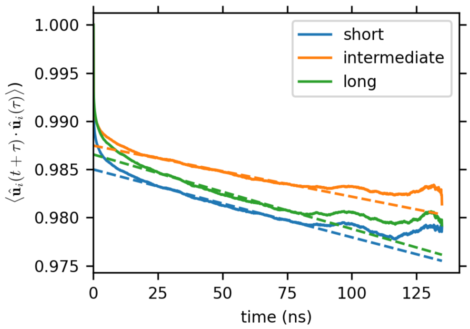

(e) 298 K

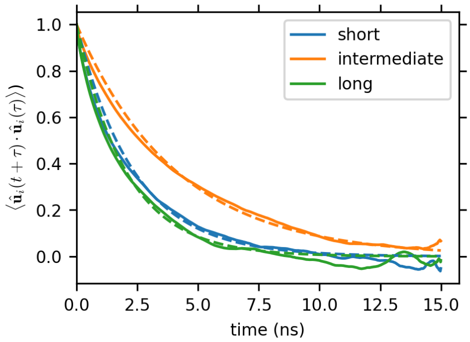

(f) 298 K (mixture)

Supplementary Figure 6: Orientational time correlation function (OTCF) versus time (solid line) of the unit vector $\hat{\mathbf{u}}_i$ aligned with each principal axis (short, intermediate, and long) of the tetracene carbon backbone of neat NODIPS-Tc at (a) 500 K, (b) 450 K, (c) 400 K, (d) 350 K, and (e) 298 K, and of (f) 50% w/w NODIPS-Tc/toluene at 298 K. The dashed line is a fit of the data to a function of the form $A_0 \exp(-2D_{\mathrm{R},i}t)$ that excludes the first tenth and last third of the data, from which the rotational diffusion coefficient $D_{\mathrm{R},i}$ was determined. In all plots except for (e), A_0 was fixed as 1, while in (e) it was allowed to vary to give the best fit in order to accommodate the sharp drop in the OTCF at short times before the rotational motion becomes diffusive.

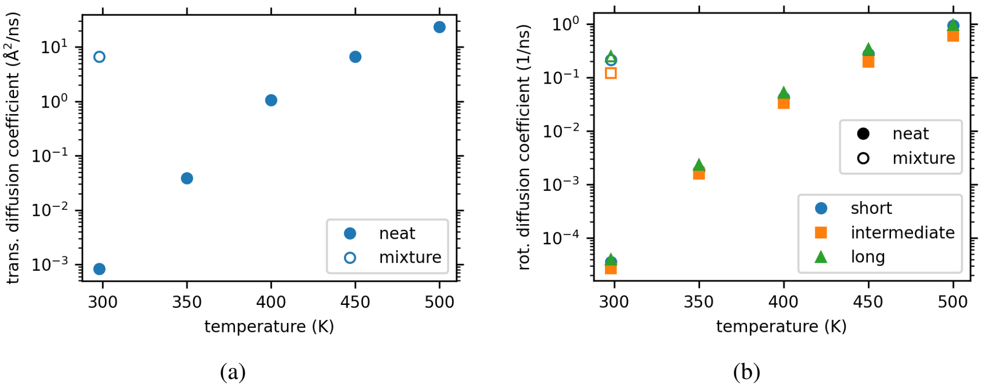

Supplementary Figure 7: (a) Translational diffusion coefficient of the center-of-mass and (b) rotational diffusion coefficient of each principal axis (short, intermediate, and long) of the tetracene carbon backbone versus temperature for neat NODIPS-Tc (filled symbols) and 50% w/w NODIPS-Tc/toluene (empty symbols).

4.3.2 Structure

The condensed-phase structure in each simulation was characterized using the radial distribution function (RDF) of the center-of-mass distance between tetracene carbon backbones of pairs of NODIPS-Tc molecules, which was calculated as

$$g(r) = \frac{\langle n(r) \rangle}{\frac{4}{3}\pi \langle \rho \rangle \left[(r + \Delta r)^3 - r^3 \right]}, \tag{14}$$

where $\langle n(r) \rangle$ is the average number of NODIPS-Tc molecules whose centers-of-mass are within a distance range r to $r + \Delta r$ of a tagged molecule at the origin and $\langle \rho \rangle$ is the average number density of NODIPS-Tc molecules in the system.

The RDFs of the neat NODIPS-Tc system at different temperatures are shown in Supplementary Figure 8a, while the RDFs of neat NODIPS-Tc and the 50% w/w NODIPS-Tc/toluene mixture are compared in Supplementary Figure 8b. From the RDFs in Supplementary Figure 8a, the structure of neat NODIPS-Tc is very similar at all temperatures, with the main difference being a narrowing of the peaks with decreasing temperature. Thus, even though it was not possible to fully equilibrate the system at 298 K due to the low molecular mobility at this temperature, we can be reasonably confident that the structure at the higher temperatures, where full equilibration was possible, extrapolates without major structural reorganization to this lowest temperature, which would be consistent with the experimental observation that neat NODIPS-Tc is an amorphous liquid at room temperature.

The room-temperature RDFs of neat NODIPS-Tc and the NODIPS-Tc/toluene mixture in Supplementary Figure 8b are very similar, indicating similar structural correlations between pairs of NODIPS-Tc molecules in the two systems. The main difference is a slight reduction in the height of the first peak in the distribution around 4–5 Å and a slight increase in the height of the peak around 11 Å in the mixture, which will be discussed further below.

Structural correlations between the anisotropic NODIPS-Tc molecules in each simulation were further characterized using the angular–radial distribution function (ARDF) of the the center-of-mass distance and the cosine of the angle between each principal axis of the tetracene carbon backbones of pairs of NODIPS-Tc molecules,

$$g(r, \cos\theta_i) = \frac{\langle n(r, \cos\theta_i) \rangle}{\frac{4}{3}\pi \langle \rho \rangle \left[(r + \Delta r)^3 - r^3 \right] \Delta \cos\theta_i}, \tag{15}$$

where $\langle n(r, \cos\theta_i) \rangle$ is the average number of NODIPS-Tc molecules whose centers-of-mass are within a distance range r to $r + \Delta r$ and whose i-axes are within a cosine angle range $\cos\theta_i$ to $\cos\theta_i + \Delta \cos\theta_i$ of the center-of-mass and i-axis, respectively, of a tagged molecule at the origin.

The ARDFs of neat NODIPS-Tc and the NODIPS-Tc/toluene mixture at 298 K are plotted for all three principal axes in Supplementary Figures 9 and 10, respectively. Due to the symmetry of the NODIPS-Tc molecule along the short and intermediate axes, the ARDF for each of these axes is an even function of $\cos\theta_i$ and so has been plotted as a function of $|\cos\theta_i|$ instead of $\cos\theta_i$. On the other hand, asymmetry of the molecule along the long axis means that positive and negative $\cos\theta_i$ are inequivalent. In the neat NODIPS-Tc system, the orientations of tetracene backbones become uncorrelated at distances larger than around

16 Å, where the ARDFs become largely homogeneous. At distances smaller than around 8 Å, there is a strong tendency for the backbones to π-stack, as indicated by the large peak in the ARDF for the short a-axis at these distances. These π-stacked configurations can be further categorized into several distinct nearest-neighbor configurations based on the distinct peaks with significant amplitude separated by regions of very low probability in the ARDFs for the intermediate b- and long c-axes. These peaks occurs at $(r, |\cos\theta_\mathrm{a}|, |\cos\theta_\mathrm{b}|, \cos\theta_\mathrm{c})$ approximately equal to (3.8 Å, 1, 0.5, 0.5), (4.4 Å, 1, 0, 0), (5.6 Å, 1, 1, -1), and (6.5 Å, 1, 1, 1), respectively. Representative examples of these pair configurations are shown in Supplementary Figure 11, in which the angle between the π-stacked tetracene backbones is 60°, 90°, 180°, and 0° for center-of-mass separations of 3.8 Å, 4.4 Å, 5.6 Å, and 6.5 Å, respectively.

The ARDFs of the NODIPS-Tc/toluene mixture in Supplementary Figure 10 share many of the same features of those of neat NODIPS-Tc, except that the two peaks at $(r, |\cos\theta_\mathrm{a}|, |\cos\theta_\mathrm{b}|, \cos\theta_\mathrm{c}) =$ (3.8 Å, 1, 0.5, 0.5) and (4.4 Å, 1, 0, 0) in the neat system merge into a single ridge with diminished probability relative to that of the (5.6 Å, 1, 1, -1) peak, with the latter peak signicantly broadened over a larger range of distances in the mixture. The (6.5 Å, 1, 1, 1) peak due to parallel π-stacked backbones is also greatly diminished compared with the (5.6 Å, 1, 1, -1) peak due to anti-parallel π-stacked backbones. In addition, the enhanced peak at around 11 Å in the RDF of the mixture corrresponds to an enhancement of configurations in which the long axes (tetracene backbones) of the two molecules are aligned ($\cos\theta_\mathrm{c} = 1$), while the short and intermediate axes are orthogonal ($\cos\theta_\mathrm{a} = \cos\theta_\mathrm{b} = 0$), i.e. a NODIPS moiety on one of the molecules separates the two tetracene backbones. Such configurations may be accommodated by the presence of the toluene solvent in this system.

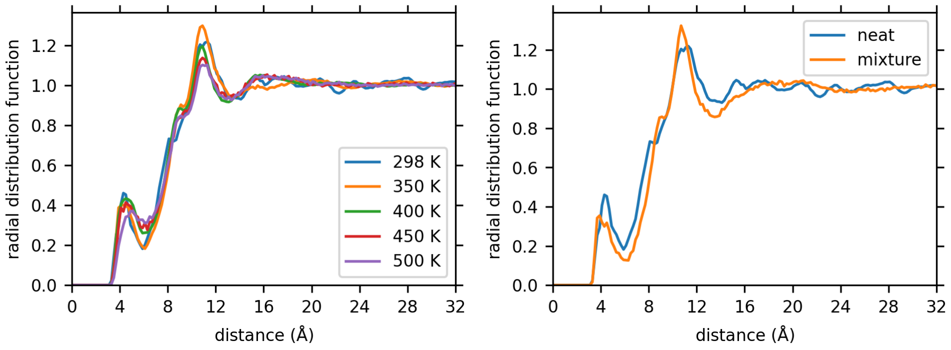

(a) (b)

Supplementary Figure 8: Radial distribution function (RDF) of the center-of-mass distance between tetracene carbon backbones of pairs of NODIPS-Tc molecules in (a) neat NODIPS-Tc at various temperatures and (b) neat NODIPS-Tc and 50% w/w NODIPS-Tc/toluene at 298 K.

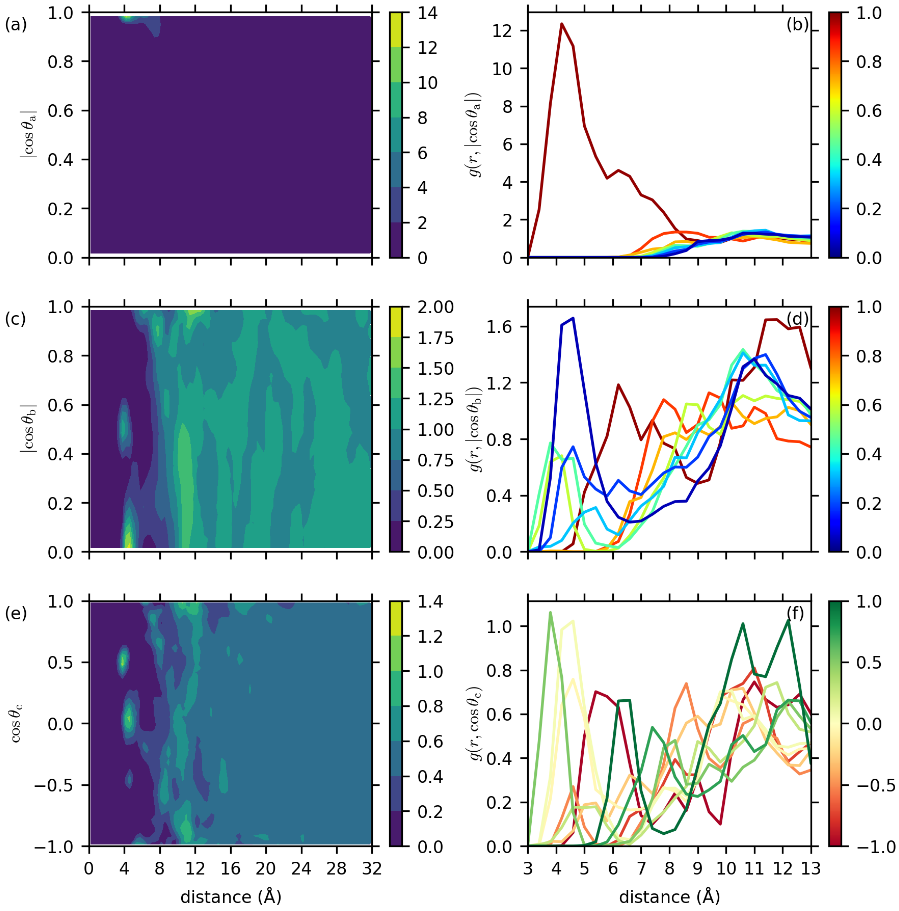

Supplementary Figure 9: Angular radial distribution function (ARDF), $g(r, \cos\theta_i)$, of the center-of-mass distance r and the cosine of the angle θ_i between principal axes of tetracene carbon backbones of pairs of NODIPS-Tc molecules for neat NODIPS-Tc at 298 K, depicted as (a, c, e) 2D contour plots and (b, d, f) 1D slices versus distance at fixed angle, for θ_i the angle between the (a, b) short axes ($i = \mathrm{a}$), (c,d) intermediate axes ($i = \mathrm{b}$), and (e,f) long axes ($i = \mathrm{c}$). Due to the symmetry of the NODIPS-Tc molecule along the short and intermediate axes, the ARDF for each of these axes is an even function of θ_i and so has been plotted as a function of $|\cos\theta_i|$. The surface in (a, c, e) is colored by the value of the ARDF, while the lines in (b, d, f) are colored by the value of $|\cos\theta_i|$. Note that there is significant noise in the ARDFs because of the low mobility of this sytem, which limits sampling of the 2D distribution on the time scale of the simulation.

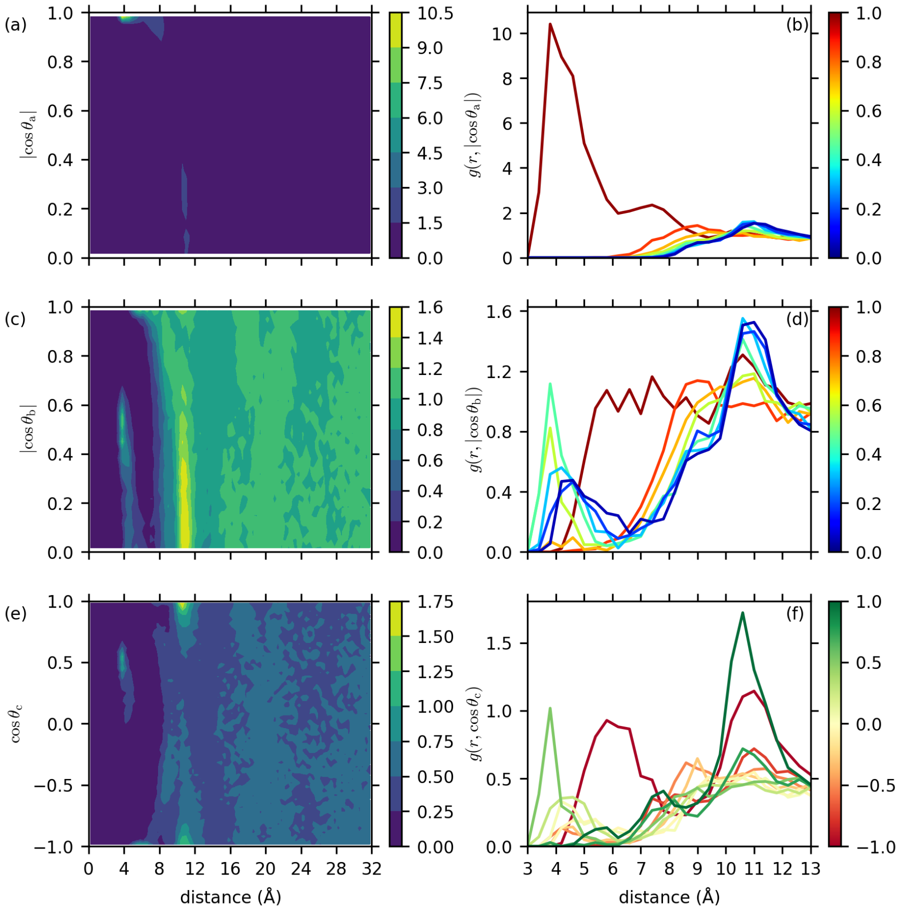

Supplementary Figure 10: Angular radial distribution function (ARDF), $g(r, \cos\theta_i)$, of the center-of-mass distance r and the cosine of the angle θ_i between principal axes of tetracene carbon backbones of pairs of NODIPS-Tc molecules for 50% w/w NODIPS-TC/toluene at 298 K, depicted as (a, c, e) 2D contour plots and (b, d, f) 1D slices versus distance at fixed angle, for θ_i the angle between the (a, b) short axes ($i = \mathrm{a}$), (c,d) intermediate axes ($i = \mathrm{b}$), and (e,f) long axes ($i = \mathrm{c}$). Due to the symmetry of the NODIPS-Tc molecule along the short and intermediate axes, the ARDF for each of these axes is an even function of θ_i and so has been plotted as a function of $|\cos\theta_i|$. The surface in (a, c, e) is colored by the value of the ARDF, while the lines in (b, d, f) are colored by the value of $|\cos\theta_i|$.

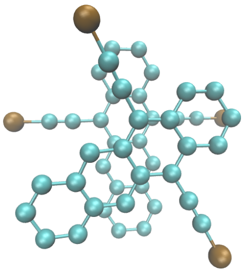

(a) 3.8 Å, 1, 0.5, 0.5 (top)

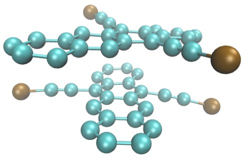

(b) 3.8 Å, 1, 0.5, 0.5 (side)

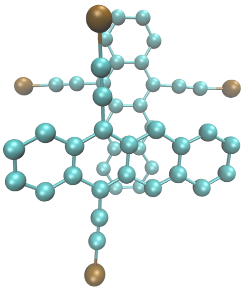

(c) 4.4 Å, 1, 0, 0 (top)

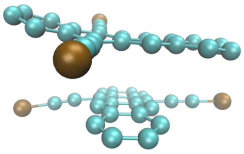

(d) 4.4 Å, 1, 0, 0 (side)

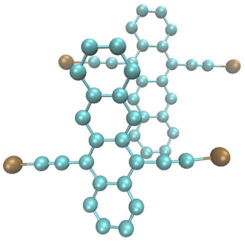

(e) 5.6 Å, 1, 1, -1 (top)

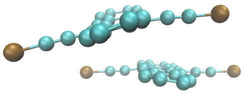

(f) 5.6 Å, 1, 1, -1 (side)

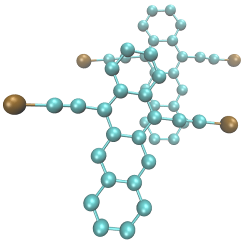

(g) 6.5 Å, 1, 1, 1 (top)

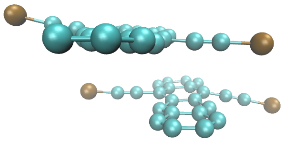

(h) 6.5 Å, 1, 1, 1 (side)

Supplementary Figure 11: Representative examples of distinct nearest-neighbor pair configurations in simulation of neat NODIPS-Tc at 298 K corresponding to the peaks in the ARDF in Supplementary Figure 9 at $(r, |\cos\theta_\mathrm{a}|, |\cos\theta_\mathrm{b}|, \cos\theta_\mathrm{c})$ approximately equal to (a,b) (3,8 Å, 1, 0.5, 0.5), (c,d) (4.4 Å, 1, 0, 0), (e,f) (5.6 Å, 1, 1, -1), and (g,h) (6.5 Å, 1, 1, 1) viewed from the top (a,c,e,g) and side (b,d,f,h). For clarity, only non-hydrogen atoms of the tetracene backbone and silylethynl moeities are shown.

5 Detailed synthetic procedure

5.1 Synthesis of n-octyl(diisopropyl)silylacetylene (NODIPS acetylene)

NODIPS acetylene was prepared as previously reported.[9]

5.2 Synthesis of 5,12-bis((diisopropyl(octyl)silyl)ethynyl)tetracene (NODIPS-Tc)

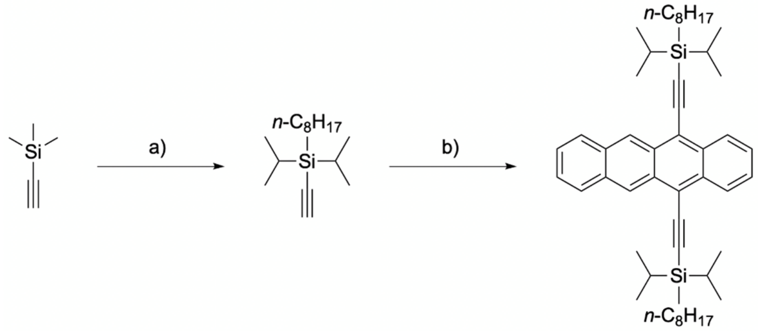

Supplementary Figure 12: Synthesis of NODIPS-Tc. Conditions: a) 1. n-BuLi, THF, 0˘25°C, 30 min., then n-C_8H_{17}(i-Pr)2SiCl, rt, 12 h., 2. K_2CO_3, CH_3OH, rt, 2 h.; b) 1. n-BuLi, THF, 0°C, 1 h., then 5,12-naphthacenequinone, rt, 18 h., 2. $SnCl_2{\cdot}2H_2O$, 10% aq. HCl, rt, 3 h.

n-Butyl lithium (0.83 mL, 1.33 mmol, 1.6M in hexanes) was added dropwise to a solution of n-octyl(diisopropyl)silylacetylene (0.347 g, 1.37 mmol) in dry THF (7 mL) at 0°C under an argon atmosphere. The resulting solution was stirred for 1 hour, followed by the addition of 5,12-naphthacenequinone (0.100 g, 0.39 mmol). The solution was warmed to rt and stirred for 18 hours under argon. To this solution was added tin(II) chloride dihydrate (0.883 g, 3.91 mmol) and 10% aqueous HCl solution (5 mL) and the mixture was stirred for 3 hours. The solution was partitioned between hexanes (20 mL) and water (20 mL) and the aqueous layer was further extracted with hexanes (3×10 mL). The combined organic layers were dried with magnesium sulfate, filtered and concentrated under reduced pressure. The crude product was purified using silica gel column chromatography with hexanes as the eluent. NODIPS-Tc was obtained as a red oil (0.709 g, 0.97 mmol, 71%).

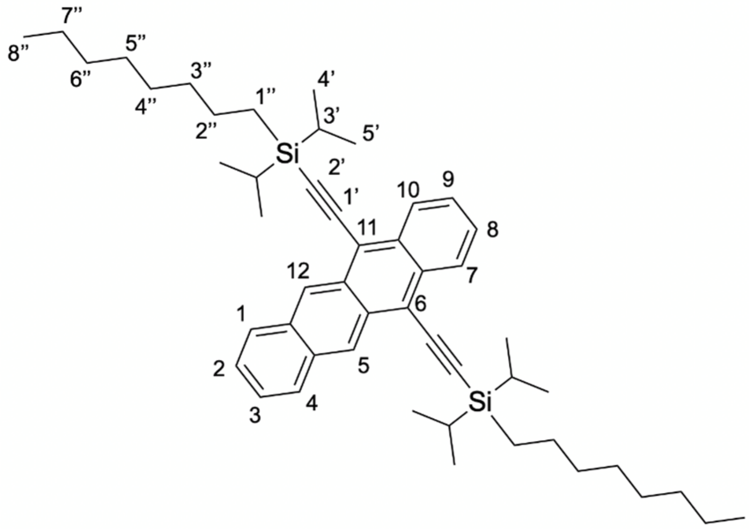

1H NMR (400 MHz, $CDCl_3$) δ 9.28 (s, 2H, H-5, H-12), 8.61 (dd, J = 6.8, 3.2 Hz, 2H, H-7, H-10), 8.02 (dd, J = 6.5, 3.2 Hz, 2H, H-1, H-4), 7.54 (dd, J = 6.8, 3.2 Hz, 2H, H-8, H-9), 7.46 (dd, J = 6.6, 3.1 Hz, 2H, H-2, H-3), 1.75 – 1.63 (m, 4H, H-2”), 1.52 – 1.42 (m, 4H, H-3”), 1.35 – 1.20 (m, 44H, H-3’, H-4’, H-5’, H-4”, H-5”, H-6”, H-7”), 0.94 – 0.80 (m, 10H, H-1”, H-8”).

13C NMR (100 MHz, $CDCl_3$) δ 132.8 (C-6a, C-10a / C-4a, C-12a), 132.3 (C-4a, C-12a / C-6a, C-10a), 130.4 (C-5a, C-11a), 128.7 (C-1, C-4), 127.6 (C-7, C-10), 126.8 (C-8, C-9), 126.4 (C-5, C-12), 126.1 (C-2, C-3), 118.7 (C-6, C-11), 106.3 (C-2’), 103.9 (C-1’), 34.1 (C-3”), 32.1 (C-4” / C-5” / C-6” / C-7”), 29.55 (C-4” / C-5” / C-6” / C-7”), 29.48 (C-4” / C-5” / C-6” / C-7”), 25.0 (C-2”), 22.8 (C-4” / C-5” / C-6” / C-7”), 18.8 (C-4’ / C-5’),* 18.5 (C-4’ / C-5’),* 14.2 (C-8”), 12.2 (C-3’), 10.5 (C-1”).

*The methyl groups of isopropyl moieties are known to be inequivalent in compounds where the isopropyl is attached to an asymmetric centre (with respect to the isopropyl).[10, 11]

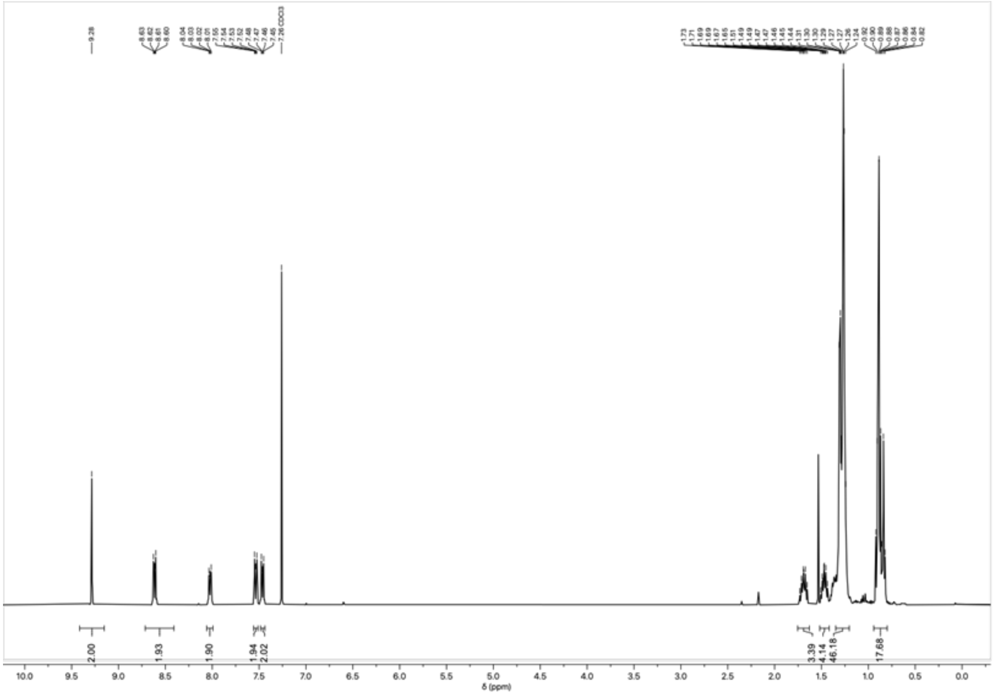

Supplementary Figure 13: ^{1}H NMR (400 MHz, $CDCl_3$) of NODIPS-Tc.

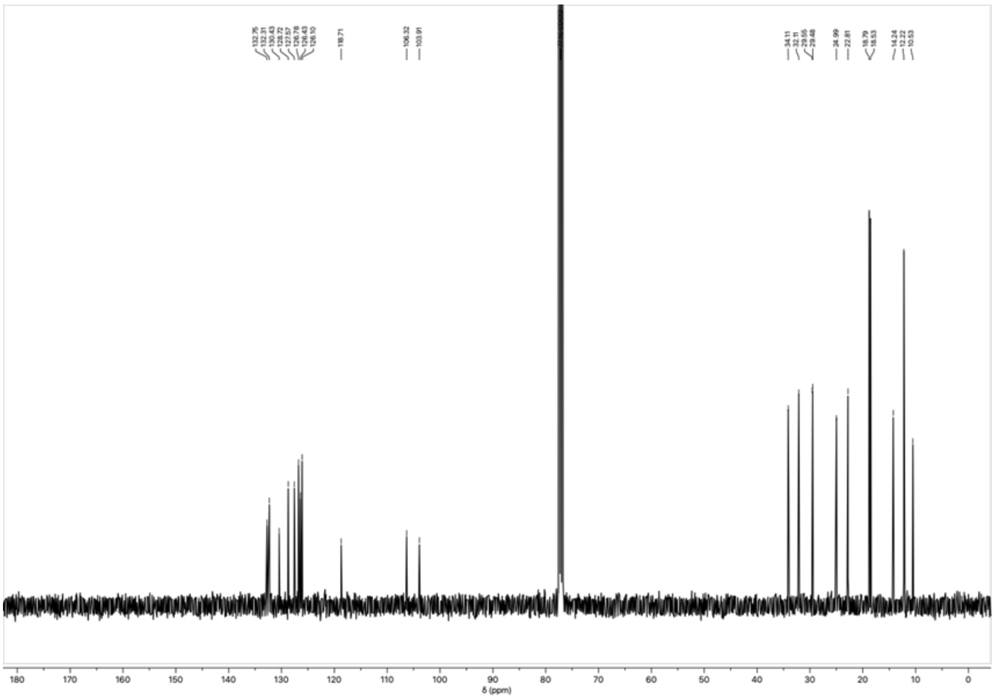

Supplementary Figure 14: ^{13}C NMR (100 MHz, $CDCl_3$) of NODIPS-Tc.

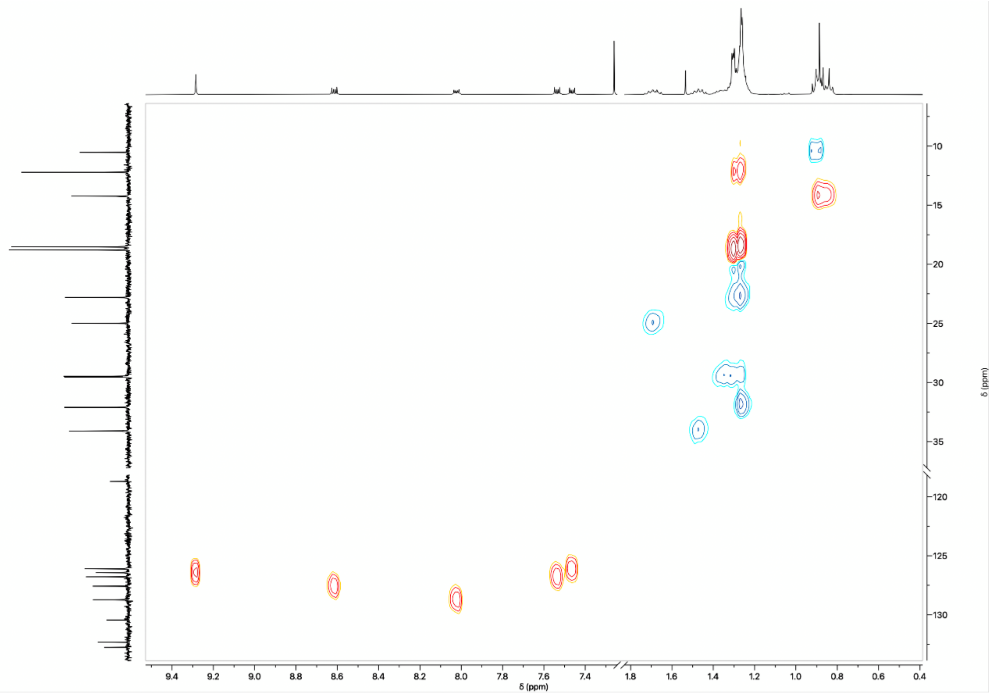

Supplementary Figure 15: HSQC NMR (400 MHz, $CDCl_3$) of NODIPS-Tc.

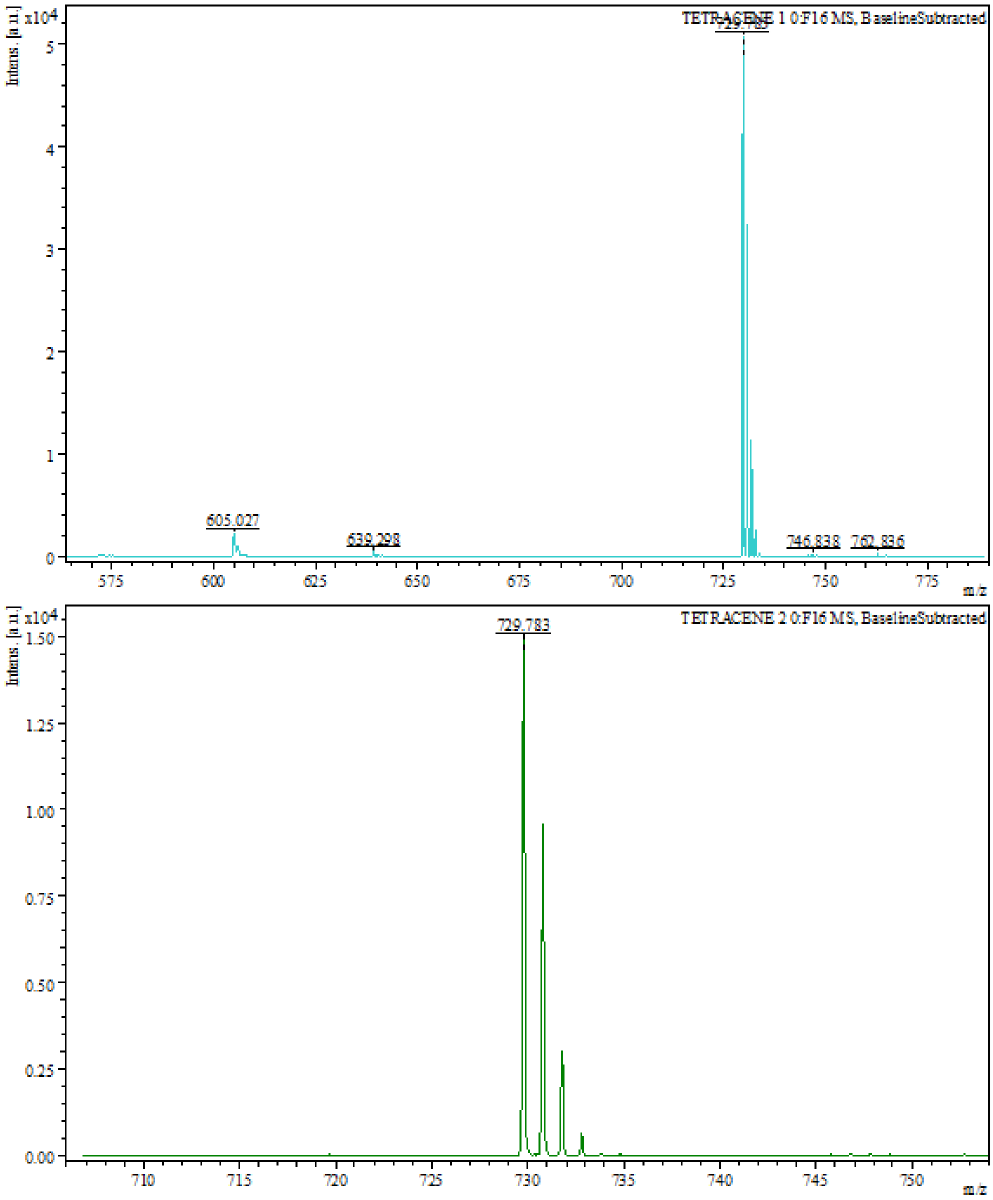

Supplementary Figure 16: MALDI-TOF of NODIPS-Tc.

6 Integrated Magnetic Photoluminescence

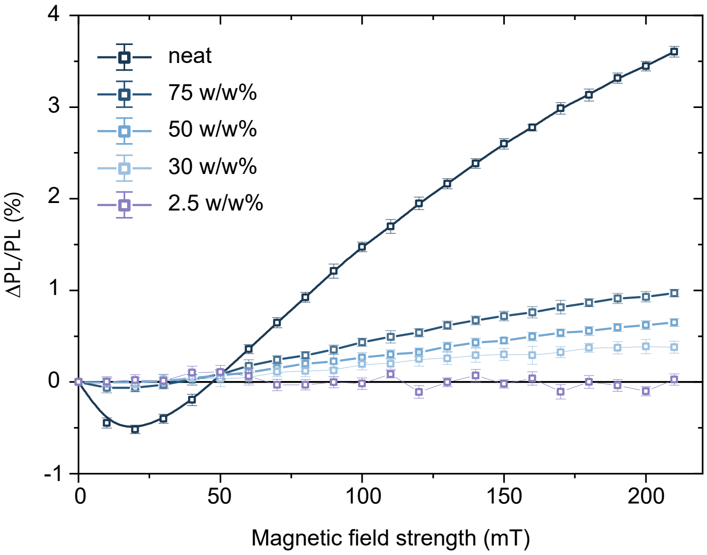

Supplementary Figure 17: Integrated magneto photoluminescence for various concentrations of NODIPS-tetracene. The error bars in the plots represent the standard error of the mean after 20 repeat measurements. Error arises due to small fluctuations in the laser diode power.

7 van ’t Hoff Plot

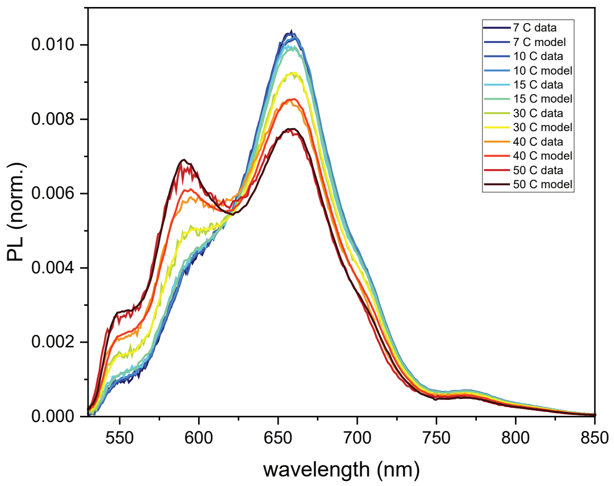

Supplementary Figure 18: Normalised spectra of Epoch 2 as a function of temperature and modelled spectra in terms of combinations of σ_{S}, σ_{TT} and σ_{Ex}.

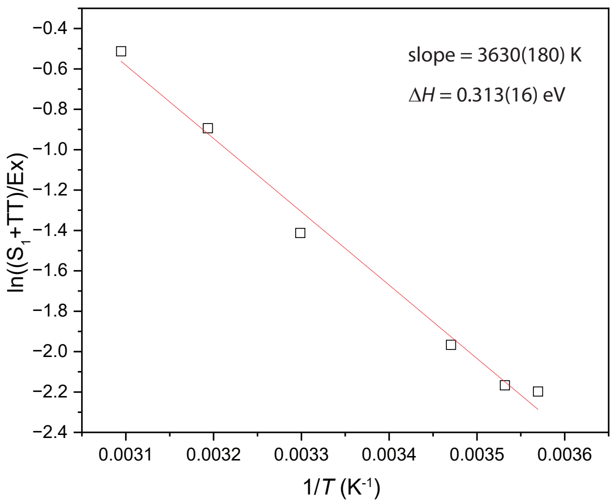

Supplementary Figure 19: Plot of the logarithm of the ratio of the sum of S_1 and $^1(\mathrm{TT})$ components to the $^1\mathrm{Ex}$ component against inverse temperature for the spectra of Epoch 2. The linear fit indicates that the excimer is 0.31 eV below the other singlet states.

Supplementary References

[1] Glasstone, S., Laidler, K. & Eyring, H. *Theory of Rate Processes* (McGraw-Hill, New York, 1941).

[2] Jorgensen, W. L., Maxwell, D. S. & Tirado-Rives, J. Development and testing of the OPLS all-atom force field on conformational energetics and properties of organic liquids. *J. Am. Chem. Soc.* **118**, 11225–11236 (1996).

[3] Jorgensen, W. L. & Tirado-Rives, J. Molecular modeling of organic and biomolecular systems using BOSS and MCPRO. *J. Comput. Chem.* **26**, 1689–1700 (2005).

[4] Jorgensen, W. L. & Tirado-Rives, J. Potential energy functions for atomic-level simulations of water and organic and biomolecular systems. *Proceedings of the National Academy of Sciences* **102**, 6665–6670 (2005).

[5] Jewett, A. I. *et al.* Moltemplate: A tool for coarse-grained modeling of complex biological matter and soft condensed matter physics. *J. Mol. Biol.* **433**, 166841 (2021).

[6] Rackers, J. A. *et al.* Tinker 8: Software tools for molecular design. *J. Chem. Theory Comput.* **14**, 5273–5289 (2018).

[7] Yeh, I.-C. & Hummer, G. System-size dependence of diffusion coefficients and viscosities from molecular dynamics simulations with periodic boundary conditions. *J. Phys. Chem. B* **108**, 15873–15879 (2004).

[8] Linke, M., Köfinger, J. & Hummer, G. Rotational diffusion depends on box size in molecular dynamics simulations. *J. Phys. Chem. Lett.* **9**, 2874–2878 (2018).

[9] Kumarasamy, E. *et al.* Properties of poly- and oligopentacenes synthesized from modular building blocks. *Macromolecules* **49**, 1279–1285 (2016).

[10] McFarlane, W. Chemical shift and coupling constant nonequivalence of isopropyl methyl protons in a tertiary phosphine. *Chem. Commun. (London)* 229–230 (1968).

[11] Jennings, W. B. Chemical shift nonequivalence in prochiral groups. *Chem. Rev.* **75**, 307–322 (1975).
